# Natural Compounds With Antimicrobial and Antiviral Effect and Nanocarriers Used for Their Transportation

**DOI:** 10.3389/fphar.2021.723233

**Published:** 2021-09-06

**Authors:** Diana Stan, Ana-Maria Enciu, Andreea Lorena Mateescu, Andreea Cristina Ion, Ariana Cristina Brezeanu, Dana Stan, Cristiana Tanase

**Affiliations:** ^1^ DDS Diagnostic, Bucharest, Romania; ^2^ Titu Maiorescu University, PhD Medical School, Bucharest, Romania; ^3^ Victor Babes National Institute of Pathology, Biochemistry–Proteomics Department, Bucharest, Romania; ^4^ Carol Davila University of Medicine and Pharmacy-Department of Plastic Surgery, Bucharest, Romania; ^5^ Titu Maiorescu University, Faculty of Medicine, Bucharest, Romania

**Keywords:** natural molecules, antimicrobial, antifungal, antiviral, drug delivery systems, nanocarriers

## Abstract

Due to the increasing prevalence of life-threatening bacterial, fungal and viral infections and the ability of these human pathogens to develop resistance to current treatment strategies, there is a great need to find and develop new compunds to combat them. These molecules must have low toxicity, specific activity and high bioavailability. The most suitable compounds for this task are usually derived from natural sources (animal, plant or even microbial). In this review article, the latest and most promising natural compounds used to combat bacteria, filamentous fungi and viruses are presented and evaluated. These include plant extracts, essential oils, small antimicrobial peptides of animal origin, bacteriocins and various groups of plant compounds (triterpenoids; alkaloids; phenols; flavonoids) with antimicrobial and antiviral activity. Data are presented on the inhibitory activity of each natural antimicrobial substance and on the putative mechanism of action against bacterial and fungal strains. The results show that among the bioactive compounds studied, triterpenoids have significant inhibitory activity against coronaviruses, but flavonoids have also been shown to inhibit SARS-COV-2. The last chapter is devoted to nanocarriers used to improve stability, bioavailability, cellular uptake/internalization, pharmacokinetic profile and reduce toxicity of natural compunds. There are a number of nanocarriers such as liposomes, drug delivery microemulsion systems, nanocapsules, solid lipid nanoparticles, polymeric micelles, dendrimers, etc. However, some of the recent studies have focused on the incorporation of natural substances with antimicrobial/antiviral activity into polymeric nanoparticles, niosomes and silver nanoparticles (which have been shown to have intrinsic antimicrobial activity). The natural antimicrobials isolated from animals and microorganisms have been shown to have good inhibitory effect on a range of pathogens, however the plants remain the most prolific source. Even if the majority of the studies for the biological activity evaluation are *in silico* or *in vitro*, their internalization in the optimum nanocarriers represents the future of “green therapeutics” as shown by some of the recent work in the field.

## Introduction

Natural products are an important source of new drugs or serve as templates for the development of new synthetic drugs, from anticancer therapies to antibiotics. A significant number of natural product drugs are actually produced by microbes or through their interaction with hosts (Newman and Cragg, 2020). One of the main reasons for exploring natural products with antimicrobial activity is the ever-expanding plasmid-transmitted antibiotic resistance genes and the presence of diseases (mainly respiratory and neurological) that are not covered by natural or plant-derived substances. The World Health Organization (WHO) promotes the use of medicinal herbs as remedies to support the absence of conventional treatment. Emphasis is placed on studies of bioactive compounds, their chemical composition, and the pharmacological potential of various plant species to produce compounds with lower toxicity than existing molecules. Due to their numerous benefits, natural compounds are now used to treat some diseases including microbial diseases, inflammatory processes and cancer. This is mainly due to the accessibility and good therapeutic potential of natural medicines (Boccolini and Boccolini, 2020). Plants play a major role in the world of medicine as a source of natural compounds of medicinal importance and represent the largest resource for new and highly effective drugs/therapies. BBC Research reports that the global market for herbal medicines will increase from $29.4 billion in 2017 to approximately $39.6 billion in 2022 (Patra et al., 2018).

Appropriate drug delivery system is a key segment in achieving convincing drug recovery responses. Nanotechnology has recently been considered as a means of producing carriers for certain molecules. Nanocarriers and innovative formulations play a tremendous role in enhancing the bioavailability and remedial potential of medications, achieving particular enrichment at the target site.

The present review article aims to highlight the recently discovered natural compounds with antimicrobial and antiviral properties and the optimal combination of active substances and nanocarriers to improve their properties, stability and overall efficacy. Therefore, the article consists of four main chapters: 1) Natural products with antibacterial activity - this chapter is devoted to antimicrobials isolated from natural sources with bacteriostatic and bactericidal activity on both Gram-positive and Gram-negative bacteria; 2) Natural products with antifungal activity - this deals with antifungal natural products used against some of the major human pathogens; 3) Natural products with antiviral activity - describes the recently discovered groups of compounds with antiviral activity and their mechanisms of action; 4) Nanocarriers as drug delivery systems - describes a number of nanocarrier-active substance complexes and their improvement and elective target.

The present work is very complex due to the inclusion of all human pathogenic groups (bacteria, fungi, and viruses) and agents that have recently been shown to inhibit and/or kill them. In addition, the mechanisms of action are described for most compounds/complex mixtures and encapsulated active substances. In addition to the comprehensive information on the natural active compounds and the vehicles used for their transport and controlled release, the paper also provides new avenues of research by highlighting the areas where further work is needed, such as elucidating the mechanism of action, establishing the minimum inhibitory concentration, finding the optimal nanocarrier, etc.

Most of the data discussed here are *in vitro* studies, but some systematic reviews on the use of nanocarriers in clinical trials (Dri et al., 2021), reviews on nanomedicine and the clinical application of various nanoparticles (Chang et al., 2021; Mitchell et al., 2021) can be consulted for further reading.

## Natural Products With Antibacterial Activity

**FIGURE 1:**
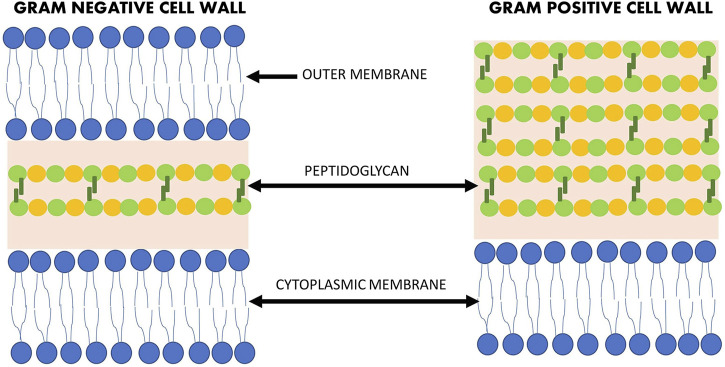
Structural differences between the Gram-negative and Gram-positive bacteria cell wall.

There are a number of natural compunds isolated from various sources (plant, animal, or microorganism) that have antibacterial activity. However, due to the structural differences between Gram-negative and Gram-positive bacteria (Figure 1), the efficacy of antimicrobial agents may vary. The prevalence of antibiotic resistance of Gram-negative strains is of serious concern, particularly in the hospital setting where immunodeficient patients are most at risk (Morris and Cerceo, 2020). However, multidrug-resistant (MDR) strains are not only found in the hospital setting, but also in our food, due to the extensive use of antibiotics in livestock for the treatment of infections, growth promotion and prophylaxis (Mateescu et al., 2014). Therefore, it is crucial to find new agents to combat these MDR strains.

### Agents of Plant Origin

Curcumin (diferuloylmethane) is a low molecular weight polyphenol (Priyadarsini, 2014) used for centuries in traditional Asian medicine for the treatment of various diseases and is isolated from the rhizome of *Curcuma longa* L. (turmeric). Numerous studies have shown that curcumin has a wide range of biological and pharmacological properties. It has been shown to be active against several important human pathogens, including strains of the genus *Staphylococcus*, *Streptococcus*, and *Enterococcus.* The activity of *Curcuma longa* L. extract against staphylococci has been investigated in several studies, and the data showed antimicrobial activity of curcumin against both methicillin-resistant *Staphylococcus aureus* (MRSA) and methicillin-susceptible *Staphylococcus aureus* (MSSA), with minimum inhibitory concentrations (MICs) in the micromolar range (Mun et al., 2013; Teow and Ali, 2015; Hung et al., 2020; Jaiswal and Mishra, 2018; Sardi et al., 2017). Moreover, a synergistic effect of *Curcuma longa* L. extract and various antibiotics (oxacillin, ampicillin, ciprofloxacin, gentamicin, amikacin, polymyxin B and norfloxacin) was observed in a strain-dependent manner, while no antagonistic effects were observed (Mun et al., 2013; Teow and Ali, 2015; Betts et al., 2016). The synergistic effects could possibly be due to the ability of curcumin to bind bacterial enzymes, thereby reducing the lysis and hydrolysis of antibiotics (Zhou et al., 2011; Teow and Ali, 2015). Free and microencapsulated curcumin showed bacteriostatic activity against *Bacillus subtilis* and *Bacillus cereus* (Jaiswal and Mishra, 2018; Praditya et al., 2019). Curcumin was also effective against *Streptococcus pyogenes* and when combined with polymyxin B, even a synergistic effect was observed (Betts et al., 2016).

*Listeria innocua* was evaluated for its sensitivity to UVA -light exposed curcumin and a synergistic effect was found even when curcumin was applied at low concentrations (de Oliveira et al., 2018). Sortase A from *Staphylococcus aureus* has been shown to be inhibited by curcumin and is also a relevant enzyme in *Staphylococcus mutans* responsible for covalent binding of the major cell surface adhesin to the cell wall, thus playing a role in biofilm formation (Hu et al., 2013).

Essential oils are complex mixtures of a variety of constituents and have long been known for their antimicrobial properties. Generally, drug combinations have been shown to be an essential feature of antimicrobial treatment as they increase activity through synergistic or additive action, reduce required doses, reduce both cost and adverse/toxic side effects, and increase the spectrum of activity (Morlock et al., 2014; Bag and Chattopadhyay, 2015).

Studies were conducted to evaluate the possible synergistic interactions on antibacterial and antioxidant activity of essential oils of some selected spices and herbs: *Laurus nobilis* L. (bay leaf), *Piper nigrum* L. (black pepper), *Coriandrum sativum* L. (coriander), *Cuminum cyminum* L. (cumin), *Allium sativum* L. (garlic), *Zingiber officinale* Roscoe (ginger), *Brassica nigra* (L.) W.D.J. Koch (black mustard), *Allium cepa* L. (onion) and *Curcuma longa* L. (turmeric), in combination. The antibacterial activity of the combination was evaluated against major Gram-positive bacterial strains like *Bacillus cereus*, *Listeria monocytogenes*, *Micrococcus luteus* and *Staphylococcus aureus* using microbroth dilution and time-kill method. From the above results, three essential oils (coriander oil, cumin oil and mustard oil) showed promising antibacterial activity against most of the bacteria studied and had an inhibition zone diameter > 11 mm (Bag and Chattopadhyay, 2015). In the antibacterial combination study, among the three combinations tested (coriander/cumin, coriander/mustard and cumin/mustard), only the coriander/cumin combination showed synergistic interaction against the Gram-positive bacteria (Bag and Chattopadhyay, 2015). To confirm the synergistic antibacterial activity of coriander-cumin combination, a time-kill assay was performed which showed that coriander-cumin combination reduced the bacterial activity. Chemical analysis revealed that linalool from coriander seed oil and p-coumaric acid from cumin seed oil were the bioactive compounds responsible for both synergistic antibacterial activities (Chernestove et al., 2014).

Essential oils extracted from *Origanum vulgare* L. (oregano) have high antimicrobial properties and can act synergistically in combination with other types of oils. In addition, the bioactive properties and composition of oregano essential oil may vary depending on the geographical area, species, or time of collection. In bacteria, the cell membrane is a very important target for oregano essential oil components, such as terpenoids, which could interfere with the phospholipid bilayers of the cytoplasmic membrane (Gumus et al., 2010). Notwithstanding these differences, carvacrol and thymol are the common volatile compounds responsible for the antimicrobial properties of oregano oil (Rodriguez-Garcia et al., 2016). These are phenolic monoterpene compounds that account for about 78–85% of oregano oil and are particularly attractive to cell membrane structures due to their lipophilic nature. These two compounds are able to dissolve the outer membrane of bacteria and release the lipopolysaccharide components, which increases the permeability of adenosine triphosphate in the cytoplasmic membrane and consequently alters the passive permeability of the cell (Guarda et al., 2011).

The antibacterial properties of honey are well documented. The main compound responsible for the antibacterial effect of honey is described as hydrogen peroxide (H_2_O_2_), which is produced mainly during the oxidation of glucose catalyzed by the bee enzyme glucose oxidase. Hydrogen peroxide alone is commonly used in high concentrations (0.8–8 M) as a disinfectant for medical equipment in hospitals. The antimicrobial activity of H_2_O_2_ has been demonstrated against several medically important bacterial species, including *Staphylococcus* spp., *Streptococcus* spp. and *Bacillus* spp. spores. The bactericidal effect of hydrogen peroxide is related to the accumulation of irreversible oxidative damage to membranes, proteins, enzymes and DNA (Bizerra et al., 2012).

Interestingly, the hydrogen peroxide content in different honeys can vary considerably from honey to honey (Brudzynski et al., 2011). The oxidizing effect of hydrogen peroxide in honey on bacterial cells may be modulated by the presence of other bioactive molecules in honey. Several natural and artificial honey samples were evaluated using *Bacillus subtilis* as bacterial reference strains to determine the correlation between hydrogen peroxide concentration and the ability to inhibit bacterial growth. In addition, treatment of honey samples with catalase allowed the evaluation of the inhibitory effect of the oxidizing activity of endogenous hydrogen peroxide on bacterial DNA (Bizerra et al., 2012).

A number of studies have reported that wasabi has antibacterial properties against foodborne pathogens such as *Escherichia coli*, *Salmonella typhimurium*, *Pseudomonas aeruginosa*, *Staphylococcus aureus*, and *Helicobacter pylori* (Dias et al., 2014). *Eutrema japonicum* (Miq.) Koidz. (Wasabi) is an edible plant that contains a variety of phytochemicals. Many studies have shown that isothiocyanates (ITC), especially allyl isothiocyanate (AITC), are responsible for the antibacterial activity as well as pungency of wasabi. Obviously, the MIC of wasabi varies with the bacteria, initial cell concentration, medium and, more importantly, wasabi source and moisture content. Meanwhile, wasabi has been shown to have bacteriostatic activity against *Staphylococcus aureus* at low concentrations and bactericidal activity at high concentrations. Wasabi has high potential to effectively control *Staphylococcus aureus* and other foodborne pathogens such as *Listeria monocytogenes*. The antibacterial property along with its natural green color, unique taste, and advantage of safely protecting food during consumption make wasabi a promising natural antibacterial plant (Lu et al., 2016).

Resveratrol (a polyphenolic compound) has been found in over 100 medicinal and edible plants such as *Reynoutria japonica* Houtt. (hu zhang), *Arachis hypogaea* L. (peanut), *Yucca shidigera* Roezl ex Ortgies (amole), *Cassia quinquangulata* Rich. (senna), *Rheum rhamponticum* L. (rhubarb), and many more (Rocha-González et al., 2008; Chachay et al., 2011). First isolated by Takaoka in 1940 from the roots of *Veratrum grandiflorum* (Maxim. ex Miq.) O.Loes. (white hellebore), the compound was later named resveratrol as it is a resorcinol derivative from *Veratrum* species (Catalgol et al., 2012).

Resveratrol shows promising antibacterial activities against major foodborne bacteria such as *Staphylococcus aureus*, *Listeria monocytogenes*, *Camplylobacter jejuni*, and *Vibrio cholerae*, possibly mediated by DNA cleavage, membrane damage, decreased cellular metabolic activity, and inhibition of cell division (Cho et al., 2013, 2015; Lee et al., 2014). The antibiofilm and antivirulence activities of resveratrol can be used as a stand-alone therapeutic alternative or as a coadjuvant to current antibiotic therapy against pathogens. It also has antifungal properties (Vestergaard and Ingmer, 2019). A study has shown that resveratrol complexed with soy protein isolate (SPI) has better physicochemical properties, but the effects of complexation on its antimicrobial properties remain to be investigated (Pujara et al., 2017).

Overall, resveratrol should be considered as an antimicrobial biopharmaceutical product and should also find significant applications in the food industry as a food preservative (Ma et al., 2018).

A major limitation of these studies is that they were conducted *in vitro*. The biochemical processing of different natural products may vary from one human cell type to another, resulting in different metabolic end products. For some products, such as honey, the preservation of oxidative activity would require local application. From this point of view, isolation of bioactive compounds delivered to the target sites by nanocarriers would be a more effective approach than the current trend of oral supplementation of antibiotic therapy.

Plants of the genus *Achillea* are described as anti-inflammatory and diuretic and show high efficacy in the treatment of inflammation, bleeding and rheumatic pain. The antioxidant and antimicrobial effects are associated with their phenolic and flavonoid content. The ethanolic extract of *Achillea abrotanoides* Vis. (yarrow) showed significant antimicrobial activity against Gram-positive bacteria *Enterococcus faecalis* in comparison with the antibiotic ampicillin (Kaczorová et al., 2021). High concentrations of the flavanone naringenin were found in the ethanolic extracts, which showed very high activity against *Enterococcus faecalis*.

In a 2015 study, extracts were obtained from 17 plant species and tested for their antimicrobial activity against fungi and bacterial strains. The plants used in this study belonged to the families: *Pinaceae* (*Abies balsamea* (L.) Mill., *Pinus banksiana* Lamb, *Tsuga canadensis* (L.) Carrière); *Ericaceae* (*Chimaphila umbellata* (L.) W.P.C.Barton, *Gaultheria hispidula* (L.) Muhl. ex Bigelow.); *Apiaceae* (*Heracleum maximum* W. Bartram); *Rubiaceae* (*Mitchella repens* L.); *Betulaceae* (*Betula papyrifera* Marshall, *Betula alleghaniensis* Britton); *Anacardiaceae* (*Rhus typhina* L.) and *Oleaceae* (*Fraxinus pennsylvanica* var. *subintegerrima* (Vahl) Fernald, *Fraxinus Americana* L., *Fraxinus nigra* Marshall, *Fraxinus quadrangulata* Michx., Fraxinus profunda (Bush) Bush, *Fraxinus mandschurica* Rupr.). The extracts were obtained by immersing different parts of the plants in 100% ethanol for 24 h and then homogenizing, filtering and collecting the filtrate. The ethanolic extracts of *Chimaphila umbellata* (L.) W.P.C. Barton (prince's pine) were found to be antimicrobial active against all tested strains including Gram-negative bacteria such as *Escherichia coli* and *Pseudomonas aeruginosa* at a concentration of 10,000 µg/ml (Vandal et al., 2015).

Another study group focused on the treatment of diarrhea caused by *Escherichia coli* with plant extracts. They selected 9 plant species including: *Hypericum roeperianum* Schimp. ex A.Rich., *Cremaspora triflora* (Thonn.) K.Schum, *Heteromorpha arborescens* (Spreng.) Cham. & Schltdl., *Pittosporum viridiflorum* Sims, *Bolusanthus speciosus* (Bolus) Harms, *Calpurnia aurea* (Aiton) Benth., *Maesa lanceolata* Forssk., *Elaeodendron croceum* (Thunb.) DC. and *Morus mesozygia* Stapf. This time, the extracts were obtained in acetone, which resulted in different extraction yields (*Hypericum roeperianum* 12%, *Maesa lanceolata* 11,12%, etc.). Among the selected microorganisms, *Escherichia coli* appeared to be the most sensitive to the acetone extracts, but *Salmonella typhimurium* and *Pseudomonas aeruginosa* also showed relatively low MIC values between 0.04 and 0.52 mg/ml (Elisha et al., 2017).

Medicinal plants like *Oxalis corniculate* L. (changeri), *Cinnamomum tamala* (Buch.-Ham.) T.Nees & C.H.Eberm. (tejpat), *Ageratina adenophora* (Spreng.) R.M.King & H.Rob. (crofton weed) and *Artemesia vulgaris* L. (mugwort) were also tested for their antimicrobial properties against some of the most common bacterial pathogens, including some multidrug resistant bacteria. The extracts were prepared using absolute methanol and successive filtration steps after the plants were previously ground to obtain a fine powder. The results showed that *Oxalis corniculata* had the most efficient antimicrobial activity against Gram-negative bacteria, with a MIC of 100 mg/ml against *Salmonella typhi* and 50 mg/ml for MDR-*Salmonella typhi*, and an MIC of 25 mg/ml for *Escherichia coli*, *Klebsiella pneumoniae* and MDR-*Citrobacter koseri* (Manandhar et al., 2019).

Pectins from *Spondias dulcis* Parkinson, also known as ambarella, were evaluated for their antimicrobial activity against reference strains of *Salmonella* spp. and clinical strains. Using the disc diffusion method, zones of inhibition ranging from 12 to 15.0 mm were determined for a pectin solution of 100 μg/ml. The MIC values determined for the different strains of *Salmonella* spp. ranged from 5.68 to 44.45 μg/ml. Most interestingly, however, treatment of *Salmonella* spp. infected mice with these extracts has been shown to prolong their lifespan (Zofou et al., 2019).

Another interesting study dealt with the evaluation of the antimicrobial activity of pelargonic acid extracted from tomatoes. They prepared pelargonic acid emulsions with different surfactants such as: Tween 80, Triton X100, Sodium Dodecyl Sulfate (SDS) and *Quillaja saponaria* Molina (quillaja). Their results showed that bactericidal activity against *Salmonella newport* occurred only when pelargonic acid emulsions containing 0.1 and 1% SDS were used, while 1% quillaja saponin emulsions were bactericidal for *Salmonella newport* and *Salmonella oranienburg* at 15.62 ± 0.00 mM and 31.25 ± 0.00 mM, respectively, and 0.1% quillaja saponin emulsions killed *Salmonella typhimurium* at a concentration of 31.25 ± 0.00 mM. They concluded that the bactericidal effect of the obtained emulsions depended on the serotype of the strain and the type of surfactant (Dev Kumar et al., 2020). A drawback of this study is the lack of data on the effect of mock micelles freed from pelargonic acid. Since both SDS and saponin are used to denature membranes and dissociate membrane proteins from lipid bilayers, their effect on the bacterial wall at the concentrations tested remains to be determined.

Propolis produced by *Trigona* spp. also showed good antimicrobial activity against *Salmonella* spp. with MIC of 0.87%. The major compound groups isolated were flavonoids and tannins which were found to be key compounds for antimicrobial activity (Hasan et al., 2011).

Essential oils extracted from plants have also been reported to show good antimicrobial activity against bacterial and fungal strains. In one study, essential oils extracted from 21 plants were tested: *Lavandula angustifolia* Mill. (lavender), *Cinnamomum zeylanicum* Flower (cinnamon), *Pinus montana* Mill. (mountain pine), *Mentha × piperita* L. (mint), *Foeniculum vulgare* Mill. (fennel), *Pinus sylvestris* L. (pine), *Satureja hortensis* L. (summer savoury), *Origanum vulgare* L. (oregano), *Pimpinella anisum* L. (anise), *Rosmarinus officinalis* L. (rosemary), *Salvia officinalis* L. (sage), *Abies alba* Mill. (silver fir), *Citrus aurantium* var. *dulcis* Hayne (bitter orange), *Citrus sinensis* (L.) Osbeck (sweet orange), *Cymbopogon nardus* (L.) Rendle (lemongrass), *Mentha spicata* L. (spearmint), *Thymus vulgaris* L. (thyme), *Carvum carvi* L. (caraway), *Thymus serpyllum* L. (wild thyme), *Ocimum basilicum* L. (basil) and *Coriandrum sativum* L. (coriander) against a number of members of the *Pseudomonadaceae* family, some of which showed resistance to a range of antibiotics including ampicillin, imipenem, meropenem and gentamicin. They concluded that the essential oil (EO) of *Cinnamomum zeylanicum* flower was most effective against *Pseudomonas* spp. with MIC values ranging from 3.125 µl/ml to 12.5 µl/ml (Kačániová et al., 2017).

The mechanism of action of the compounds presented above against Gram-negative bacteria is shown in Table 1.

**TABLE 1:** Mechanism of action of plant compounds against Gram-negative strains.

Compound	Source	MIC value range	Action mechanism
Ethanolic extract	*Chimaphila umbellate* L. Spreng., *Gaultheria hispidula* L. Muhl.	10.000 µg/ml	Unclear, possible cytoplasmic rapture (Brobbey et al., 2017)
Ethanolic extract	*Rhus typhina* L.		
Acetone extract	*Hypericum roeperianum* G.W. Schimp. ex A.Rich. var. *Roeperianum*	0.09–0.28 mg/ml	Regulation of IL-7 and stimulation of CD4^+^ and CD8^+^ lymphocytes Mwitari et al. (2013)
Acetone extract	*Cremaspora triflora* (Thonn.) K.Schum		Not reported, possibly cell wall related Elisha et al. (2017)
Acetone extract	*Heracleum maximum* Bart.		Not reported, possibly cell wall related Elisha et al. (2017)
Acetone extract	*Pittosporum viridiflorum* Sims		Not reported, possibly cell wall related Elisha et al. (2017)
Acetone extract	*Bolusanthus speciosus* (H. Bolus)*, Calpurnia aurea* (Aiton) Benth ssp. aurea		Not reported, possibly cell wall related Elisha et al. (2017)
Acetone extract	*Maesa lanceolata* Forssk		Not reported, possibly cell wall related Elisha et al. (2017)
Acetone extract	*Elaeodendron croceum* (Thunb.) DC.		Not reported, possibly cell wall related Elisha et al. (2017)
Acetone extract	*Morus mesozygia* Stapf ex A.Chev		Not reported, possibly cell wall related Elisha et al. (2017)
Methanolic extract	*Oxalis corniculate* L.	25–100 mg/ml	Polyphenols binding to bacterial proteins inhibiting adhesion and microbial growth Mukherjee et al. (2013)
Pectin	*Spondias dulcis* Parkinson	5.68–44.45 μg/ml	Putative strong oxidizer - generation of high ROS amount (reactive oxygen species) Ciriminna et al. (2020)
Pelargonic acid	*Solanum lycopersicum* L.	15.62 ± 00–31.25 ± 00 mM	Cell membrane damage and lysis Kumar (2020)
Propolis	*Trigona* spp.	0.87%	Several mechanisms: increase in cell membrane permeability; reduction of ATP production; lowering mobility, disturbance of membrane potential; stimulation of host immune system Przybyłek and Karpiński (2019)
Esential oil	*Cinnamomum zeylanicum* Nees.	3.125–12.5 µl/ml	Growth inhibition without disintegrating the outer-membrane or intracellular ATP depletion Nazzaro et al. (2013)

#### Endemic Plants Extract With Antibacterial Properties

Soltanian et al. (2020) investigated the antibacterial potential of methanol extracts from three *Acantholimon* (prickly thorn) species endemic to Iran, including *Acantholimon austroiranicum* Rech.f. & Schiman-Czeika, *Acantholimon serotinum* Rech.f. & Schiman-Czeika and *Acantholimon chlorostegium* Rech.f. & Schiman-Czeika. The methanolic extracts were tested against both Gram-positive and Gram-negative bacteria, and the antibacterial activity was significantly higher for Gram-negative bacteria. Higher inhibitory activities were observed against *Escherichia coli* and *Pseudomonas aeruginosa* and weaker antibacterial activities against *Enterococcus faecalis* and *Staphylococcus aureus*.

*Anabasis aretioides* Moq. & Coss. ex Bunge is a plant endemic to Morocco and Algeria, widely used in traditional medicine as a diuretic, antirheumatic and poison antidote. Methanolic and macerated methanol extracts of *Anabasis aretioides* were tested for their antimicrobial activity against *Proteus mirabilis*, *Bacillus subtilis*, *Staphylococcus aureus* and *Pseudomonas aeruginosa* (Senhaji et al., 2020). Due to the phenolic content of ethyl acetate extract, which has the highest zone of inhibition against *Staphylococcus aureus*, cold methanolic macerated extract and hot methanolic extract were bactericidal to *Proteus mirabilis* and *Bacillus subtilis*. Chloroform extract was also tested and showed bactericidal effect on *Bacillus subtilis.*

The endemic plant *Doronicum macrolepis* Freyn & Sint. is believed to have potential uses in the treatment of diseases such as diabetes and Alzheimer's disease (Özcan, 2020). The extracts and oils of *Doronicum macrolepis* also showed good antibacterial activity. The essential oil showed inhibitory effect on *Escherichia coli*, *Staphylococcus epidermidis*, *Enterococcus faecium*, *Yersinia pseudotuberculosis*, *Candida albican* and *Candida tropicalis*.

### Agents of Animal Origin

Arenicins are a group of peptides that have been shown to have good antimicrobial activity against Gram-negative bacteria. This group consists of 3 types: arenicin-1, arenicin-2 and arenicin-3. Arenicin-1 isolated from *Arenicola marina* (sandworm) showed potent antimicrobial activity against *Escherichia coli* and *Pseudomonas aeruginosa*, with MIC values between 1 and 2 µM (Orlov et al., 2019). Another recent study showed that a slightly modified arenicin-3 peptide had considerable antimicrobial activity even against XDR (extensive drug resistance) and MDR (multi-drug resistance) strains such as *Pseudomonas aeruginosa*, *Acinetobacter baumannii*, *Escherichia coli* and *Klebsiella pneumoniae* (Elliott et al., 2020).

Another antimicrobial peptide isolated from frog skin hemocytes is poly(glycolide-co-lactide) (PGLA), and it has been shown to prevent bacterial adhesion by causing a conformational change and elimination of bacterial pili (da Silva and Teschke, 2003).

Also derived from frogs, the antimicrobial peptide magainin has been isolated from the African clawed frog (*Xenopus laevis*). However, studies show that resistant *Escherichia coli* strains can easily develop as shown by Maria-Neto et al. (2012) who produced resistant clones after ten consecutive propagations in magainin I at 37.5 mg/L^−1^.

A well-known antimicrobial agent of animal origin is chitosan, which is derived from chitin by deacetylation. Goy et al. (2016), Verlee et al. (2017) reported that mixing culture broth with polymeric medium transiently impaired the growth rate of *Escherichia coli* and increasing chitosan concentrations (0.5–2.0 g/L) resulted in a linear tendency to reduce bacterial population.

Seroins are low molecular weight proteins found in silk produced by *Bombyx mori* (silkworm). While the antimicrobial properties of seroin 1 and 2 are well known, seroin 3 has only recently been studied. A recent study showed that seroin 1, seroin 1-N, and seroin 1-C had no bacteriostatic effect on *Escherichia coli*, seroin 2 had better antibacterial activity, and seroin 3 had stronger bacteriostatic activity against Gram-positive bacteria than against Gram-negative strains (Zhu et al., 2020).

The mechanism behind the antimicrobial properties of each animal-derived compound presented against Gram-negative strains is shown in Table 2.

**TABLE 2:** Mechanism of action of antibacterial compounds of animal origin against Gram-negative strains.

Compound	Source	MIC value range	Action mechanism
Arenicin	*Arenicola marina*	1–2 µM	Membrane interaction - Outer-membrane lesions induced by current fluctuations in the lipid bilayer Andrä et al. (2008)
PGLa	Frog skin	≥64 μM	Putative mechanism -displacement of Mg^2+^ions from the lipopolysaccharide layer and PGLa insertion
Magainin	*Xenopus laevis*	75 mg L^−1^.	Permeabilization of bacterial membranes – efflux of intracellular K+ ions Matsuzaki et al. (1997)
Chitosan	Crustacean chitin	0.5–2.0 g/L	Peptidoglycan hydrolysis Goy et al. (2016)
Seroin	*Bombyx mori*	1–20 μg (depending on seroin type and exposure time)	Growth inhibition Singh et al. (2014)

### Agents of Bacterial Origin

*Actinobacteria* represent the most important group of microorganisms producing bioactive compounds. They synthesize about two-thirds of all naturally derived antibiotics currently used in medicine and veterinary medicine, with most of these molecules coming from the genus *Streptomyces* (Barka et al., 2016; Chater, 2016). Among the new molecules reported since 2011, maclamicin is a new polyketide of the spirotetronate class with polycyclic composition derived from the endophytic *Micromonospora* spp. GMKU326 isolated in Thailand. Maclamicin exhibited potent antimicrobial activities against Gram-positive bacteria including *Micrococcus luteus*, *Bacillus subtilis*, *Bacillus cereus*, *Staphylococcus aureus* and *Enterococcus faecalis* with MIC values of 0.2, 1.7, 6.5, 13 and 13 μg/ml, respectively. Significant antibacterial activities were exhibited by lobophorin F, a new spirotetronate molecule isolated from *Streptomyces* spp. SCSIO 01127. The MIC values against *Bacillus thuringiensis*, *Staphylococcus aureus* and *Enterococcus faecalis* were 2, 8 and 8 μg/ml, respectively (Niu et al., 2011).

*Lactobacillus plantarum* B21 isolated from Vietnamese sausage (nem chua) has previously shown broad antimicrobial activity against Gram-positive bacteria, including foodborne pathogens *Listeria monocytogenes* and *Clostridium perfringens*. In this study, the antimicrobial agent was successfully identified as plantacyclin B21AG, a circular bacteriocin with 5668 Da that exhibits high thermostability, resistance to a wide pH range, proteolytic resistance, and temporal stability (Golneshin et al., 2020). Its antimicrobial spectrum of activity included killing activity against closely related *Lactobacillus* species and foodborne pathogens *Clostridium perfringens* and *Listeria monocytogenes*. This newly identified circular bacteriocin has many properties that would be desirable for food preservation.

There are a number of lactic acid bacteria, yeasts, and bacilli that can synthesize antimicrobial compounds that have potent activity against Gram-negative bacteria.

Garvicin KS, a bacteriocin produced by *Lactococcus garvieae*, has been shown to inhibit *Acinetobacter* spp. but no other Gram-negative bacteria and has a synergistic effect with polymyxin B against *Acinetobacter* spp. and *Escherichia coli* (Chi and Holo, 2018).

*Weissella hellenica*, a Gram-positive *bacillus*, produces weissellicin D, a broad-spectrum antimicrobial agent with impressive stability. This bacteriocin inhibited *Escherichia coli* and *Pseudomonas aeruginosa* and showed resistance to catalase, lipase, α-amylase, lysozyme, and glucoamylase (Chen et al., 2014). Another active peptide that also inhibits the growth of Gram-negative bacteria was isolated from an *Enterococcus faecalis* strain from human saliva (Cui et al., 2020).

Sonorensin, an antimicrobial peptide produced by *Bacillus sonorensis*, is able to kill non-multiplying *Escherichia coli* cells with similar efficacy to nisin, another antimicrobial peptide produced by *Lactococcus lactis* (Chopra et al., 2015).

A strain of *Lactobacillus plantarum* isolated from kombucha (a fermented bubble tea) produced a bacteriocin-like peptide called SLG10 with antimicrobial activity against both Gram-positive and Gram-negative bacteria. Stability studies showed that the peptide retained its antimicrobial properties at 37°C for 14 days and at 4°C for 2 months and was stable at pH values between 2.0 and 7.0. The MIC values obtained for SLG10 against the susceptible bacteria ranged from 16–32 µg/ml (Pei et al., 2020).

Studies show that patients suffering from neutropenic cancer are more susceptible to infections caused by Gram-negative bacteria. A recent study showed that enterocin 12 A produced by vaginal *Enterococcus faecium* can inhibit MDR Gram-negative bacteria such as *Salmonella enterica*, *Shigella flexneri*, *Escherichia coli*, and *Vibrio cholerae*, as well as the proliferation of cancer cells (Sharma et al., 2021).

Recently, a new approach for screening bacteriocins was presented based on a bioinformatics algorithm. Yount et al. (2020) used the BACIIα algorithm to identify and classify bacteriocins with high specificity. In this way, they were able to detect all bacteriocin families of the II type (with a specificity of 86%), whereupon they identified putative bacteriocins that subsequently showed broad-spectrum antimicrobial activity against a range of human pathogens. The selected putative bacteriocin sequences belonged to different microorganisms such as: *Bacillus thuringiensis*, *Eubacterium rectale*, *Bacillus cereus* and *Enterococcus pallens*.

The mechanism of action of the above presented compounds against Gram-negative bacteria is presented in Table 3.

**TABLE 3:** Mechanism of action of antibacterial compounds of bacterial origin against Gram-negative strains.

Compound	Source	MIC value range	Action mechanism
Garvicin KS	*Lactococcus garvieae*	2560 BU/ml	Not reported – possible membrane disruption Telke et al. (2019)
Weissellicin D	*Weissella hellenica*	Not reported	Not reported
Sonorensin	*Bacillus sonorensis*	∼50 μg/ml	Possible increase of membrane permeability Chopra et al. (2015).
SLG10	*Lactobacillus plantarum*	16–32 μg/ml	Possible increase of membrane permeability, massive loss of K^+^ ions Pei et al. (2020)
Enterocin 12 A	*Enterococcus faecium*	Not reported	Increase of membrane permeability Sharma et al. (2021)

## Natural Products With Antifungal Activity

Of the millions of fungal species, only a few hundred are pathogenic to humans, resulting in local or systemic fungal infections, with over 150 million severe cases of fungal infections worldwide. Invasive fungal infections are life-threatening and occur mainly in patients with weakened immune status, either due to diseases such as HIV or leukemia, or due to medical interventions such as chemotherapy or long stays in intensive care units, as in the case of SARS-CoV-2 infected patients (Blackwell, 2011; Koehler et al., 2014; Kainz et al., 2020).

In recent decades, the development of new compounds with antifungal activity has proven to be a real challenge, especially since the fungal cell is structurally and metabolically similar to the human cell, which increases the risk of toxicity and adverse effects of antifungal drugs. Currently, the five major classes of antifungal drugs used in the clinic are pyrimidines, allylamines, azoles, polyenes, and echinocandins. Two of these classes of antifungals, the polyenes and the echinocandins, are derived from natural products - bacteria and fungi, respectively. In particular, the mechanisms of action target the metabolism of ergosterol, the fungal analog of cholesterol and an essential component of the fungal cell membrane, but also other eukaryotic cell types, including human cells (Table 4). Since there are few fungal-specific targets, antifungal drug discovery is problematic and leads to the emergence of resistant fungi. In addition to intrinsic antifungal resistance (e.g., multidrug-resistant *Candida auris*), there are many other mechanisms that fungi use to resist drug therapy that have been described in the literature (Nett and Andes, 2016; Robbins et al., 2016; Perfect, 2017; Zheng and Wang, 2019; Howard et al., 2020).

**TABLE 4:** Mechanism of action of currently used antifungal agents and modes of resistance.

Antifungal class	Mecanism of action	Modes of resistance
Polyenes	Disrupts fungal cell membrane function by binding to ergosterol, forming pores through which H+ and K+ ions can escape, leading to cell death.	Decreased access to target - sequestration of ergosterol
		Increased filamentation Howard et al. (2020)
Pyrimidine analogues	Inhibits fungal RNA and DNA syntesis	Decreased drug uptake due to cytosine permease
		Decreased cytosine deaminase activity Chandra et al. (2009)
Allylamines	Inhibits squalene epoxidase, which is required for ergosterol synthesis, and causes squalene, a substance toxic to fungal cells, to accumulate intracellularly, leading to cell death.	Mutations in the squalene epoxidase gene result in failure to block ergosterol biosynthesis Rudramurthy et al. (2018)
Azoles	Inhibition of the enzyme lanosterol 14α-demethylase, which is essential for the formation of ergosterol present in the fungal cell membrane.	Overexpression and mutations of targeted proteins (ERG11)
		Upregulation of efflux pumps in cell membranes
		Lanosterol 14α-demethylase mutations Rocio et al. (2020)
Echinocandins	Interrupts fungal cell wall synthesis by inhibiting the β-1,3 glucan enzyme complex	Mutations induced in targeted proteins (Fks1 and Fks2)
		Howard et al. (2020)

Researchers are attempting to develop new antifungal agents by optimizing agents within existing drug classes or by targeting novel mechanisms such as the calcineurin pathway, glycosylphosphatidylinositol (GPI) anchor biosynthesis, or farnesyltransferase inhibition. Other approaches include the use of molecules with other medicinal purposes (such as statins, which also interfere with cholesterol metabolism), the study of natural products with antifungal activity, biosynthesis, and the use of chemical probing methods or compound libraries (Amirkia and Heinrich, 2015; Robbins et al., 2016; Nami et al., 2019; Howard et al., 2020; Morio et al., 2020; Parthasarathy et al., 2020; Tavakkoli et al., 2020).

**FIGURE 2:**
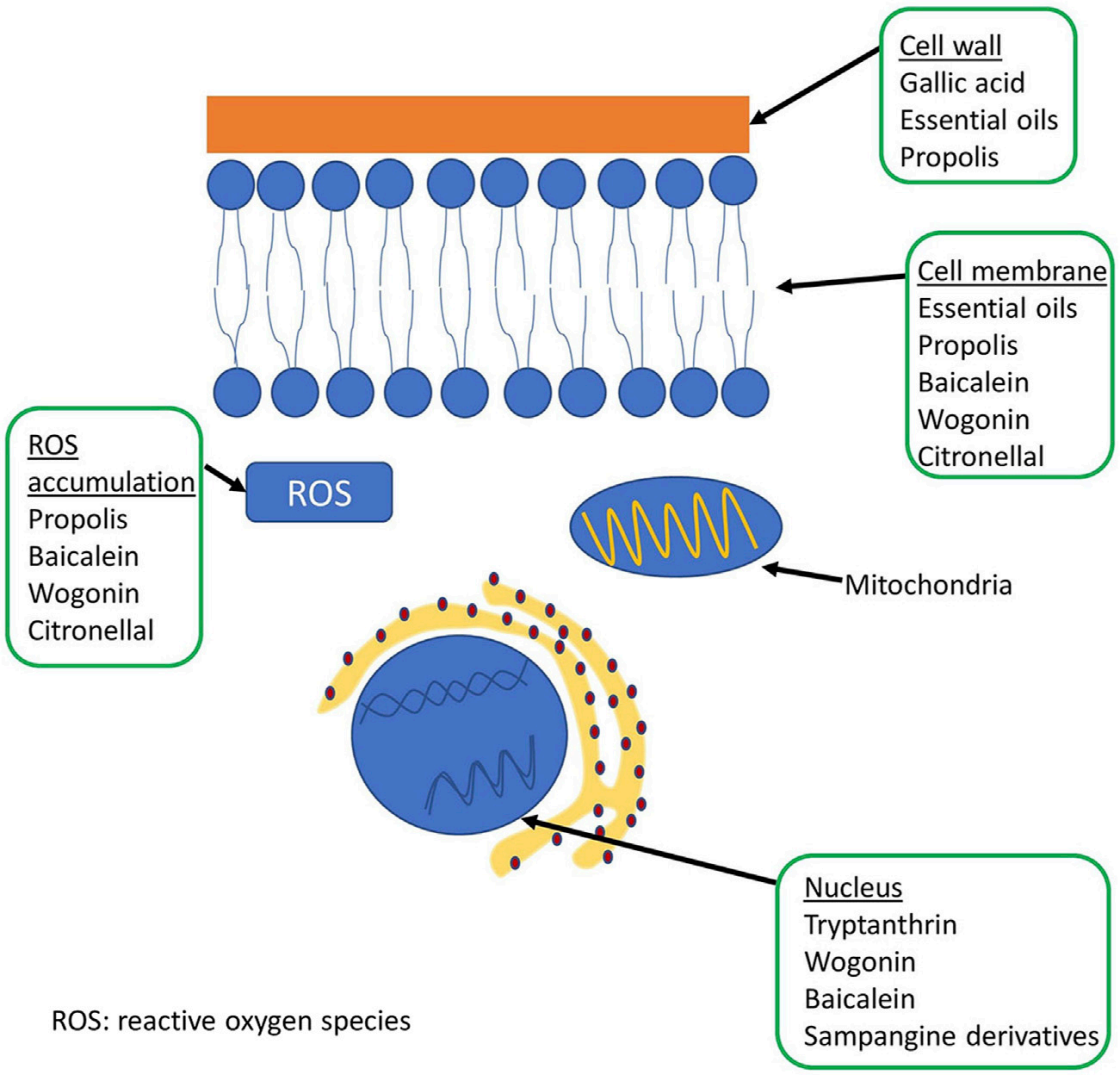
Representation of the fungal cell structure and place of action of some natural compounds.

A schematic representation of the fungal cell and the site of action of some natural compounds is shown in Figure 2. Table 5 shows the mechanisms of action of some natural compounds from different sources with antifungal activity.

**TABLE 5:** Mechanism of action of antifungal compounds of natural origin.

Compound	Source	MIC value range	Action mechanism
Methanolic extract	*Eucalyptus globulus* Labill.	0.1875–1.5 mg/ml	Not reported Natália et al. (2015)
Methanolic extract	*Juglans regia* L.	≥1.5 mg/ml	Not reported Natália et al. (2015)
Ethanolic/methanolic extract	*Lawsonia inermis* L.	5 mg/ml	Not reported Fahad et al. (2019)
Ethanolic/methanolic extract	*Withania somnifer* (L.) Dunal	5 mg/ml	Not reported Fahad et al. (2019))
Ethanolic/methanolic extract	*Curcuma longa* L.	5 mg/ml	Not reported Fahad et al. (2019)
Ethanolic/methanolic extract	*Cymbopogon citratus* (DC.) Stapf	5 mg/ml	Not reported Fahad et al. (2019)
Ethanolic/methanolic extract	*Zingiber officinale* Roscoe	5 mg/ml	Not reported Fahad et al. (2019)
Essential oil	*Cinnamomum verum* J.Presl	3.39–10.45 μg/ml	Not reported Débora et al. (2015)
Essential oil	*Melaleuca alternifolia* (Maiden & Betche) Cheel	4.84–20.03 μg/ml	Affects membrane/cell wall Débora et al. (2015)
Essential oil	*Laurus nobilis* L.	250–500 μg/ml	Inhibits cell wall formation
			Affects membrane ionic permeability (Peixoto et al., 2017)
Aqueous extract	*Buchenavia tomentosa* Eichler	0.2–25 mg/ml^−1^	Not reported Guilherme et al., (2015)
Honey	Commercially available honey	8–14.25 μg/ml	Not reported Débora et al. (2015)
Propolis extract	Brazilian propolis	837–1,675 μg/ml	Alteration in cell wall and membrane
			Inhibition of germination and filamentation
			ROS accumulation Jakeline et al. (2020)
Baicalein	*Scutellaria baicalensis* Georgi root	0.03–0.12 mM	Plasmatic membrane disintegration
			DNA fragmentation Accumulation of ROS
			Changes at the ultrastructural level Da et al. (2019)
Wogonin	*Scutellaria baicalensis* Georgi root	0.06–0.23 mM	Plasmatic membrane disintegration
			DNA fragmentation Accumulation of ROS
			Changes at the ultrastructural level Da et al. (2019)
Carvacrol	Plant extract	63–205 μg/ml	Anti-adherence activity
			Anti-proteinase activity Shaban et al. (2020)
Thymol	Plant extract	156–625 μg/ml	Anti-adherence activity
			Anti-proteinase activity Shaban et al. (2020)
Eugenol	Plant extract	321–1,250 μg/ml	Not reported Shaban et al. (2020)
Methyl eugenol	Plant extract	625–1,250 μg/ml	Not reported Shaban et al. (2020)
Gallic acid	*Punica granatum* L.	12.5–100 μg/ml	Inhibition of ergosterol biosynthesis
			Reduction of squalene epoxidase activity Li et al. (2017)
Citronellal	*Cymbopogon* spp. extract	1.2 mg/ml	Disrupts cell membrane homeostasis
			Oxidative and genotoxic effects via ROS formation
			Inhibits biofilm formation Singh et al. (2016)
Formyl phloroglucinol meroterpenoids	*Eucalyptus robusta* Sm.	1.53–50 μg/ml	Not reported Shang et al. (2019)
Magnoflorine	Plant extract	50–100 μg/ml	Inhibition of α-glucosidase activity Kim et al. (2018)
Tryptanthrin	Plant extract. Cell culture	2–8 μg/ml	Affects cell tolerance to stress like high temperature
			Induces cell cycle arrest in the G1/S phase
			Modifies AhR-dependent gene expression Lin et al. (2020)
Sampangine derivatives	Plant extract	16–0.031 μg/ml	Cell death
			Induces cell cycle arrest in the G1/S phase Li et al. (2019)
Cinnamaldehyde	Plant extract	100 μg/ml	Not reported Kim et al. (2018)
Berberine	Plant extract	100 μg/ml	Not reported Kim et al. (2018)
Teasaponin	Plant extract	64 μg/ml	Hyphal and biofilm suppression via intracellular cAMP Li et al. (2020)
Resveratrol	Plant extract	20 μM	Fungal cell apoptosis via caspase-dependent pathway Lee and Lee (2015)

### Agents of Plant Origin

In a study of *Scutellaria baicalensis* Georgi root extracts (aqueous, alcoholic, acidic) on several fungal species, the results showed marked antifungal activity against some dermatophytes, *Aspergillus fumigatus* and *Candida albicans*. In this extract, two components were identified to exhibit potent antifungal activity: baicalein and wogonin. While baicalein showed antifungal activity against all fungi tested, wogonin had no effect on *Candida albicans*. SYTOX^®^, TUNEL, SEM and TEM were used to analyze the mechanism of action of the two bioactive compounds. The results showed that baicalein and wogonin lead to plasma membrane disintegration, DNA fragmentation, accumulation of reactive oxygen species (ROS) and other changes at ultrastructural level (Da et al., 2019).

In another study investigating the effect of ten hydroalcoholic plant extracts against nineteen *Candida* species, four extracts showed antifungal activity. Plant extracts of *Juglans regia* L. (walnut), *Eucalyptus globulus* Labill. (eucalyptus), *Pterospartum tridentatum* L Willk. (carqueja) and *Rubus ulmifolius* Schott (Elm-leaved blackberry) showed a zone of inhibition between 9 and 21 mm, with strains *Candida glabrata*, *Candida albicans*, *Candida parapsilosis* and *Candida tropicalis* being the most sensitive to antifungal activity (Natália et al., 2015).

A 2019 study tested fifteen alcoholic plant leaf extracts against *Candida albicans* isolated from the oral cavity compared to standard antifungal treatment. The results showed that extracts of *Lawsonia inermis* L. (henna), *Withania somnifera* (L.) Dunal (ashwagandha), *Curcuma longa* L. (turmeric), *Cymbopogon citratus* (DC.) Stapf (lemongrass) and *Zingiber officinale* Roscoe (ginger) have the best inhibitory activity against *Candida* spp. with diameters of inhibition zones ranging from 30 to 11 mm at different concentrations (Fahad et al., 2019).

The aqueous extract of *Buchenavia tomentosa* Eichler (mirindiba) showed antifungal activity against some *Candida* species while it exhibited low cytotoxicity. Bioactive molecules in *Buchenavia tomentosa* extract such as gallic acid, corilagin and ellagic acid showed inhibitory effect on *Candida glabrata*, at low MIC values (0.004–0.25 mg/ml^−1^). Ellagic acid had antifungal activity against *albicans* and non-*albicans* species (MIC 0.004–1 mg/ml^−1^), while corilagin had antifungal activity against all non-*albicans* species (MIC 0.004–0.5 mg/ml^−1^). The antifungal activity of ellagic acid and vitexin should be further investigated as it showed promising results (Guilherme et al., 2015).

The antifungal activity of Brazilian propolis was studied on different *Candida albicans* isolates by analyzing growth kinetics, germination and filament formation, cell wall and membrane evaluation, and mutagenic potential. The results show that the propolis extract has fungistatic and fungicidal activity (MIC 3,350–6,700 μg/ml) and inhibits germination and filamentation. Calcofluor white assay was used to evaluate the effect on cell wall and membrane and the results showed that the extract leads to rupture of cell wall. Two other studies conducted using Brazilian and Iranian propolis extracts showed similar results in terms of MIC (Capoci et al., 2015; Jakeline et al., 2020).

The antifungal activity of teasaponin (saponin) extracted from tea has been studied against *Candida albicans*, and the results showed that it suppressed filament formation via intracellular cAMP and also caused inhibition of biofilm formation (Li et al., 2020).

Resveratrol, a natural polyphenolic compound with antifungal, anti-inflammatory, antihypertensive, and antiallergic effects, has been shown to induce apoptosis in *Candida albicans* cells, increase the formation of ROS, and cause mitochondrial dysfunction (Houillé et al., 2014; Lee and Lee, 2015).

Gallic acid is a natural polyphenol with anti-inflammatory and antibacterial properties and is used in several traditional medicine systems to treat bacterial and fungal infections. The antifungal activity of gallic acid was tested *in vivo*, where intraperitoneal infusions (80 mg/kg) increased the cure rate in mice with systemic fungal infections with *Candida albicans*. *In vitro* antifungal activity was studied on various dermatophyte strains and *Candida* spp. The mechanism of action was investigated by HPLC and immunosorbent assay, which revealed that gallic acid reduced the activity of sterol 14α-demethylase P450 and squalene epoxidase in the membrane of *Trichophyton rubrum* (Li et al., 2017).

The study of magnoflorin, an alkaloid present in some plants such as *Magnolia officinalis* Rehder & E.H.Wilson (magnolia), *Acorus calamus* L. (calamus) or *Tinospora cordifolia* (Willd.) Hook.f. & Thomson (gilo) has shown potential antifungal, anticancer, antioxidant and antiviral activity. The antifungal effect of magnoflorin may be attributed to the inhibition of α-glucosidase activity, which is required for normal cell wall composition and virulence of *Candida albicans*. In the same study, the antifungal effects of cinnamaldehyde and berberine were investigated, and the results showed promising effects on some *Candida* strains. In another paper published in 2021, the antifungal activity of magnoflorin on *Trichophyton* spp. was investigated with an MIC of 62.5 μg/ml. This bioactive compound has been shown to inhibit hyphal growth and alter mycelial morphology. In this study, magnoflorin showed no effects on cell wall integrity, but destroyed the fungal cell membrane by increasing nucleic acid leakage, decreased the enzymatic activity of squalene epoxidase and CYP51, and decreased the ergosterol content in hyphae (Kim et al., 2018; Luo et al., 2021).

Tryptanthrin is a natural alkaloid with indoloquinazoline content and can be chemically synthesized or obtained from natural sources such as plant extracts or various cell cultures, including yeast. This natural alkaloid has been shown to have anticryptococcal activity and a synergistic effect when used in combination with calcineurin inhibitors, but may not be as efficient *in vivo* due to poor blood-brain barrier penetration (Ramandeep et al., 2017; Lin et al., 2020).

Sampangine derivatives (a natural alkaloid) have been studied for the treatment of cryptococcal meningitis using mouse models. Among all the compounds, compound 9a showed great antifungal potential against *Cryptococcus neoformans* with MIC of 0.031 μg/ml, also it inhibited biofilm formation at a concentration of 0.062 μg/ml. This compound exhibited good blood-brain barrier permeability and stability, but has low water solubility, and further studies need to be conducted to determine its pharmacokinetics and pharmacodynamics (Li et al., 2019).

Another bioactive compound, citronellal (terpenoid), present in *Cymbopogon* spp. (lemongrass) essential oil, was tested for its antifungal activity against *Candida albicans* and non-*albicans* species. This monoterpenoid was active against both *Candida albicans* and non-*albicans* species at an MIC of 1.2 mg/ml. The main mechanism of action was disruption of membrane function and inhibition of virulent properties (Singh et al., 2016).

In an extensive research work on 82 commercially available essential oils, their effect on *Aspergillus niger*, *Candida albicans* and *Cryptococcus neoformans* was investigated. Using GC-MS analysis to examine the composition of the essential oils, the researchers found 157 different major constituents, 114 of which were unique. The major constituents of the essential oils were sesquiterpenoids, sesquiterpenes, sesquiterpenols, decane derivatives, monoterpenes, monoterpenoids, and santalol derivatives. Of all the pathogens, *Candida neoformans* showed the highest sensitivity, with MIC values ≤ 160 ppm, while *Candida albicans* was the least sensitive. *Cedrus atlantica* (Endl.) Manetti ex Carrière (Atlantic cedar) essential oil showed the best MIC for *Candida albicans* (80 ppm) and *Santalum spicatum* showed the lowest MIC for *Candida neoformans* (Powers et al., 2019).

The antifungal activity of essential oil from *Laurus nobilis* L. (laurel) was tested against some *Candida* spp. and the mechanism of action was investigated. The results showed that all strains tested were susceptible and that the addition of an osmotic protector (0.8 M sorbitol) and the presence of exogenous ergosterol increased the MIC values, suggesting that laurel essential oil affects the cell wall. *Laurus nobilis* essential oil also inhibited biofilm formation and reduced virulence of *Candida* spp. (Peixoto et al., 2017).

In another study, the antifungal effect of essential oils of *Cinnamomum verum* J. Presl (cinnamon) and *Melaleuca alternifolia* (Maiden & Betche) Cheel (tea tree) and honey on 30 *Candida* spp. isolated from HIV-positive patients was investigated. In this study, the essential oils had a stronger fungistatic effect on the fungal pathogens than honey. Analysis of the fungicidal effect showed that *Cinnamomum verum* essential oil inhibited 93.3% of *Candida* species, while *Melaleuca alternifolia* essential oil inhibited 73.3% (Débora et al., 2015).

In a recent study, the effect of currently used antifungal agents against multidrug-resistant *Candida auris* was enhanced by the addition of monoterpene phenols. The results showed that carvacrol was the most potent phenol with antifungal activity when used alone or in combination with currently used antifungal drugs by inhibiting proteinase production and adherence ability (Shaban et al., 2020).

In another study, quercetin and rutin, two flavonoids, acted only as adjuvants for amphotericin B, lowering its MIC against *Cryptococcus neoformans* from 0.25 μg/ml to 0.125 μg/ml and 0.0625 μg/ml, respectively (Oliveira et al., 2016).

The antifungal activity of miconazole was enhanced when it was used in combination with magnoflorin (alkaloid); the MIC of miconazole decreased from 3.125 μg/ml to 1.5625 μg/ml (Kim et al., 2018).

Twelve formylphloroglucinol meroterpenoids from the leaves of *Eucalyptus robusta* Sm. were tested against *Candida* spp. (+)-Eucalrobusone X showed the strongest antifungal activity against *Candida albicans* with a MIC of 10.78 μg/ml, while eucalrobusone U showed antifungal activity against *Candida glabrata* with a MIC of 1.53 μg/ml. Compounds 4 and 6 showed a MIC value of less than 10 μg/ml when tested against *Candida glabrata* (Shang et al., 2019).

The antifungal activity of ethanolic extract of *Achillea abrotanoides* (Vis.) Vis against *Candida albicans* was investigated in a recent study. The results showed good antifungal activity, moreover, the ethanolic extract showed stronger activity against *Candida albicans* than the antifungal drug (Kaczorová et al., 2021).

Antifungal activity against *Candida albicans* was also investigated in another recent study using the ethanolic extract of *Aconitum lycoctonum* L. (Karalija et al., 2021). *Aconitum* species exhibit many pharmacological properties, especially anti-inflammatory and antimicrobial activity. The ethanolic flower extract and petroleum ether extracts of leaves and stems of *Aconitum lycoctonum* L. showed significant antioxidant activity due to phenolic compounds present in the extracts. The antifungal activity results of ethanolic extract showed strong antimicrobial activity due to high concentration of flavonoids and phenolic acid in the extract composition.

### Agents Derived From Microorganisms

Microbial secondary metabolites are a great source of bioactive molecules, and in a recent research paper, the authors used a high-throughput chemogenetic screening approach to discover silent gene clusters that may be a source of new natural products. In this study, the cytotoxins etoposide and ivermectin were used as potent inducers. Using this method, the researchers activated a silent gene cluster in *Streptomyces albus* and were able to isolate and characterize 14 new molecules, one of which is a new potential antifungal agent. Acyl-surugamide A, one of the 14 new metabolites produced by sur activation in *S. albus*, showed antifungal properties with an IC_50_ of 3.5 μM against *Saccharomyces cerevisiae* (Xu et al., 2017; Pham et al., 2019).

Meijiemycin, a giant linear polyene polyol isolated from the *Streptomycetes* strain, inhibited hyphal growth by inducing ergosterol aggregation and interfering with the structure of the fungal plasma membrane. This compound was discovered using a genome-directed approach and tested against *Candida albicans* isolated from the urinary tract, showing a MIC of 12 μg/ml (Low et al., 2020).

In another research paper, the authors used a silkworm model infected with *Aspergillus fumigatus* to screen a natural product library for antifungal agents. The library was composed of microorganisms that inhibit the spread of *Aspergillus fumigatus in vitro*. Of the 4,997 fungal strains tested, only 310 were selected as potential producers of anti-*Aspergillus* compounds and tested *in vitro* and *in vivo* using silkworm and infected mouse models. The results showed the identification of a new compound with antifungal activity (ASP2397) that protects silkworm larvae and mice from aspergillosis (Nakamura et al., 2017).

Compounds from cyanobacteria such as peptides, alkaloids, terpenoids, and macrolides have known antimicrobial activity. In a study published in 2015, the bioactive compounds of cyanobacteria were tested for their antifungal activity and the macrolide scytophycin and the glycolipopeptide hassallidin were detected in *Anabaena* spp. *Anabaena cylindrica*, *Nostoc* spp. and *Scytonema* spp. In addition, the authors discovered in three cyanobacterial strains - *Fischerella* spp., *Scytonema hofmanni*, and *Nostoc* spp. - several unidentified antifungal compounds that require further characterization (Shishido et al., 2015).

Isolated lactic acid bacteria from honey showed antifungal activity against some *Candida species*. The sensitivity of *Candida* spp. to the antifungal compounds varied depending on the species and the active compounds evaluated, and the inhibitory area ranged from 0 to 22 mm (Bulgasem et al., 2016).

In another study published in 2019, the lipopeptide jagaricin produced by a bacterial fungal pathogen showed fungicidal effects against human pathogenic fungi by disrupting membrane integrity. They also found that deletions in the ERG5 and ERG6 genes did not affect the susceptibility of *Candida albicans* and *Candida glabrata* to jagaricin (Fischer et al., 2019).

Occidiofungin is another lipopeptide with antifungal properties and is produced by *Burkolderia contaminans*. It can be administered intravenously, intraperitoneally, subcutaneously, or topically and has minimal toxic effects on mammals. This natural compound has a novel mechanism of action and different cellular targets (binding to the cytoskeleton) than currently available classes of antifungal agents. Studies suggest that occidiofungin has affinity for actin-rich regions in the cell and leads to apoptosis (Ravichandran et al., 2020).

Enfumafungin is a triterpenoid isolated from *Hormonema carpetanum*. Its antifungal activity is due to inhibition of beta-1,3-glucan synthase and alteration of cell wall metabolism. It was first obtained from the *Hormonema fungus*, which occurs on the leaves of *Juniperus communis* L. (juniper), and was tested *in vivo* on a mouse model infected with *Candida albicans*. It exhibited weak antifungal activity compared to amphotericin B and pneumocandin A0. In another study MK -3118, a derivative of enfumafungin, was shown to have high antifungal activity *in vitro* against *Candida* and *Aspergillus* species (MIC 0.03–16 μg/ml), including wild-type and echinocandin-resistant isolates containing mutations in the FKS gene(s). This derivative is currently in a phase2 clinical trial (Peláez et al., 2000; Jiménez-Ortigosa et al., 2014; Wring et al., 2017).

## Natural Products With Antiviral Activity

For most viral infections, treatment consists of synthetic drugs, including agents such as neuraminidase inhibitors (zanamivir and oseltamivir), channel blockers (amantadine and rimantadine), viral polymerase inhibitors (favipiravir and baloxavir-marboxi), ribavirin, lopinavir-ritonavir, interferon, corticosteroids, and many others (Omrani et al., 2021). Their low efficacy and the development of viral resistance led to an increasing interest in natural products as an important source of antiviral and inhibitory activities. Plant extracts offer a wide range of compounds including flavonoids, alkaloids, phenolic acids, terpenes, coumarins, lignans and proteins. These complex structures have significant inhibitory potential and could be used as complementary therapies against viral infections. Recent studies have focused on the potential antiviral activity of compounds extracted from plants against viruses such as: Coronavirus, Influenza, Dengue and Herpes simplex.

Scientists found that some natural compounds in extracts of aromatic herbs and medicinal plants have antiviral properties against coronaviruses and can inhibit viral replication (Boukhatem and Setzer, 2020). The most studied groups of compounds and complex mixtures with antiviral activity include: triterpenoids, alkaloids, phenols, flavonoids, various plant extracts and honey.

### Agents of Plant Origin

Plants rich in triterpenoids have been found to have important inhibitory activity against coronaviruses. The extract from the leaves of *Euphorbia neriifolia* L. (Indian spurge) contained sugars, tannins, flavonoids, alkaloids and triterpenoid saponins. There are several types of isolated triterpenoids from *Euphorbia neriifolia* L. and the structural features of each may influence the antiviral activity on H-CoV. Thirteen of the isolated compounds were evaluated for their anti-HCoV activity; they are as follows: 3β-acetoxy-friedelan, friedelin, glutinol acetate, epitaraxerol, epitaraxeryl acetate, taraxeryl acetate, damarenediol II -acetate, cabraleadiol monoacetate, 3β-simiarenol, 24-oxocycloart-25-ene-3β-ol, (23Z)-cycloart-23-ene-3β,25-diol, cycloeucalenol and 29-norcycloartanol. The acetyl group of 3β-acetoxy-friedelane compound negatively affects the antiviral activity compared to 3β-friedelanol. Epitaraxerol, a friedelan derivative from the triterpenoids of *Euphorbia neriifolia* L., exhibits the most significant antiviral activity (Chang et al., 2012).

Li et al. (2005) have demonstrated the antiviral activity of the alkaloid fractions of *Lycoris radiata* (L'Hér.) Herb. (red spider lily) against SARS-associated coronavirus. The researchers separated the total alkaloid compounds into four fractionated samples, A, B, C and D, by RP-HPLC and evaluated their activity against SARS-CoV. They found that only fraction B has inhibitory activity against this type of coronavirus. Moreover, they confirm that lycorine, an active constituent of the alkaloid, is the active constituent of *Lycoris radiata* with potent antiviral activity against SARS-CoV.

The potential antiviral activity of aqueous and hydro-methanolic extracts from the barks and leaves of three different mulberry species: *Morus alba* L. var. *alba*, *Morus alba* var. *rosea* and *Morus rubra* L. against human coronavirus 229E was investigated by Inès Thabti et al. (2020). Among the aqueous stem bark extracts, *Morus alba* var. *rosea* has the highest inhibition percentage (36%), while the hydro-methanolic stem bark extract of mulberry species has a higher inhibition percentage of viral infectivity than the aqueous extracts (from 37 to 45%). The inhibitory activity of leaf extracts of *Morus* spp. is higher than that of stem bark extracts; for aqueous extracts, the inhibitory percentage ranges from 38.54% (*Morus alba* var. *rosea*) to 48% (*Morus alba* var. *alba*) and for hydromethanolic leaf extracts from 67% (*Morus rubra*) to 100% (*Morus alba* var. *alba*).

In a recent study, the inhibitory activity of ethanolic extract of *Sambucus javanica* subsp. chinensis (Lindl.) Fukuoka (*Sambucus Formosana Nakai*) stem against human coronavirus NL63 (HCoV-NL63) was investigated in comparison with some markers of its phenolic acid components, such as caffeic acid, chlorogenic acid, coumaric acid, ferulic acid and gallic acid. The ethanolic stem extract showed low cytotoxicity, decreased cytopathic effect in HCoV-NL63 infected cells, depending on the concentration. Caffeic acid extracted from the phenolic acid components of *Sambucus Formosana Nakai* had the highest anti-HCoV-NL63 potency, the half-maximal inhibitory concentration (IC_50_) for virus yield was 3.54 μM, for plaque formation IC_50_ = 5.40 μM and for virus attachment IC_50_ = 8.10 μM, followed by chlorogenic acid with a value for virus yield IC_50_ = 43.45 μM and coumaric acid with IC_50_ = 71.48 μM. The antiviral mechanism of caffeic acid against HCoV-NL63 may be due to the fact that caffeic acid directly targets and interferes with the binding interaction of HCoV-NL63 with heparan sulfate proteoglycans (co-receptor) and angiotensin-converting enzyme 2 (ACE2) as receptor) on the cell surface (Weng et al., 2019).

Joshi et al. (2020) investigated the inhibitory activity of some natural products against SARS-CoV-2 using Mpro and ACE2. They chose the non-structural protein Mpro as a target to stop viral replication and the ACE2 receptor to block the entry of SARS-CoV-2 into host cells. The study was carried out using a molecular docking approach and the results showed that 7 of the 11 plants tested, namely *Phaseolus vulgaris* L. (bean), *Ocimum gratissimum* L. (African basil), *Syzygium aromaticum* (L.) Merr. & L.M. Perry (clove), *Curcuma longa* L. (turmeric), *Piper longum* L. (Indian long pepper), *Inula helenium* L. (elecampane) and *Artemisia absinthium* L. (absinthe) contain secondary phytochemicals, especially flavonoids, with lower binding energy and higher affinity for Mpro and ACE2 compared to SARS-CoV-2.

SARS-CoV-2 contains two proteases, papain-like protease (PLpro) and chymotrypsin-like protease (3CLPro). Using a molecular docking method, scientists identified a series of compounds extracted from three different species of rhizomes that showed inhibitory activity on SARS-CoV-2 PLpro (Goswami et al., 2020). However, among the three rhizome species (*Alpinia officinarum* Hance, *Zingiber officinale* Roscoe and *Curcuma longa* L.), only five compounds from *Alpinia officinarum* (compounds 16, 13, 45, 36 and 22) and three from ginger (compounds 8-gingerol, 10-gingerol and 6-gingerol) showed better potential as inhibitors of PLpro with higher values of binding affinity and ligand efficiency (LE) than curcumin derivatives.

Kumar (2020) tested by molecular docking the efficacy of some natural compounds from *Ocimum sanctum* L. (Tulsi) and *Azadirachta indica* A. Juss. (Neem) against SARS-CoV-2. The phytochemicals selected for the study were rosmarinic acid, oleanolic acid, ursolic acid and methyleugenol extracted from *Ocimum sanctum* L. and azadirachtin, nimbine, epoxyazadiradione and gedunin from *Azadirachta indica* A. Juss. The results of the study indicate that all the natural products studied were effective in preventing the attachment and replication of SARS-CoV-2 virus by binding to the spike glycoprotein, RNA polymerase and/or its protease. Other natural products that have shown antiviral activity against SARS-CoV-2 replication due to inhibition of RNA polymerase or RNA-dependent proteases are the phenolic compounds from *Melia azedarach* L. (Persian lilac), *Camellia sinensis* (L.) Kuntze (tea) and from the essential oils of *Laurus nobilis* L. (laurel), *Salvia officinalis* L. (sage) and *Thuja orientalis* L. (white cedar) (Llivisaca-Contreras et al., 2021).

In recent years, researchers have focused on the antiviral activity of various compounds derived from plant extracts against SARS-CoV-2 (Khan et al., 2021). The phenolic compound coumarin, isolated from various plants such as tonka bean (*Dipteryx odorata* Aubl. Willd.) and *Cinnamomum aromaticum* Nees, has antiviral properties by blocking the protease enzyme of SARS-CoV-2. Naringenin, the predominant flavanone in fruits such as grapefruits, citrus fruits and tomatoes, acts as an inhibitor of endolysosomal two-pore channels involved in COVID -19 infections.

Among the natural options to combat SARS-CoV-2, there are several candidates worth mentioning. *Glycyrrhiza glabra* L. (licorice) contains glycyrrhizin, a saponin-like compound that interferes with the early replication stages of the SARS-CoV virus by affecting the receptor ACE -2. Similarly, 17 organosulfur compounds have been identified in the essential oil of *Allium sativum* L., the common garlic, that interact with the ACE -2 receptor. Diarylheptanoids, isolated from *Alnus japonica* (Thunb.) Steud. (alder), inhibit the activity of papain-like protease, an enzyme required for the replication process of SARS-CoV. The extract of *Houttuynia cordata* Thunb. (chameleon plant) has inhibitory effect on protease (3CLpro) and RNA-dependent RNA polymerase (RdRp) of SARS-CoV3C. Similarly, 4 natural compounds present in *Tinospora cordifolia* (Willd.) Hook.f. & Thomson (gilo), isocolumbin, magnoflorin, berberin and tinocordiside, were found to have enhanced binding efficacy for 3CLpro, RdRp, receptor binding domain and glycoprotein (Ali et al., 2021).

The antiviral activity of *Vitis vinifera* L. (grape) extracts on SARS-CoV-2 was investigated, and a strong reduction in S expression was observed at low concentrations (Zannella et al., 2021). *Vitis vinifera* leaf extract is known to be a very good antiviral agent, although it has not yet been studied for these properties. The identified phenolic extracts from *Vitis vinifera* extracts, most of which are derived from quercetin, inhibit replication in the early stages of SARS-CoV-2 infection, with the antiviral effect occurring by blocking interaction with the cell membrane. Different analytical conditions were used to study the antiviral properties of the leaf extracts: the simultaneous addition of extract and virus to cells ("co-treatment"); virus incubated with extract and then added to cells ("virus pre-treatment"); infected cells treated with the extract ("post-treatment"); and healthy cells treated with the extract and then infected with the virus ("cell pre-treatment"). It was found that the most effective conditions were co-treatment and virus pre-treatment.

Japanese apricot, *Prunus mume* (Siebold) Siebold & Zucc. also inhibits herpes simplex virus replication due to its high content of polyphenols. Some infected HEp-2 cells were exposed to the phenolics from the ethanolic extract of Japanese apricot to determine their effect on the viral replication process. The final yield of both types of herpesviruses decreased exponentially with increasing concentration of phenols, and HSV-2 was less sensitive than HSV-1 (Nishide et al., 2019).

Flavonoid fractions of various natural extracts are also important for anti-herpetic activity. El-Toumy et al. (2018) investigated the anti-herpetic activity of twenty-five Egyptian plant species, but only two of them showed good properties against herpes simplex virus type 1: *Euphorbia cooperi* N.E.Br. ex A. Berger (small candelabra tree) and *Morus alba* L. (white mulberry). In addition, the extracts of *Euphorbia cooperi* and *Morus alba* were fractionated to obtain flavonoids, which have been reported to have inhibitory effects on herpes virus. They found that flavonoids such as gallic acid, 7-O- galloyl-catechin and curcumin had the strongest antiviral activity against HSV-1. Although flavonoids such as quercetin and kaempferol were mentioned in the literature for their antiviral properties, they showed lower values for anti-herpetic activity.

*Veronica* species is a medicinal plant used as a therapeutic agent for the treatment of influenza, respiratory diseases, cancer and diabetes. Flavonoid and phenolic compounds in *Veronica* genus have antiviral, anti-inflammatory and antioxidant properties (Nazlic et al., 2020). Sharifi-Rad et al. (2018) investigated the potential antiviral activity of *Veronica persica* Poir. (speedwell) on both herpes simplex virus (HSV) types. Six different fractions of ethanolic extract of *Veronica persica* were tested with a stepwise methanol (MeOH) gradient: 0, 20, 40, 60, 80, and 100%, but the most active was the 80% MeOH extract, which showed the lowest IC_50_ values and significant selectivity and efficacy against both HSV-1 and HSV-2 viruses. Among the two types of herpes viruses, the extract of *Veronica persica* exhibited higher activity against HSV-1 as compared to HSV-2, which was significantly increased when used concomitantly with aciclovir (antiviral drug for the treatment of herpes simplex virus infections).

Ellan et al. (2019) investigated the antiviral activity of five extracts from edible and medicinal mushrooms: *Lignosus rhinocerotis* (Cooke) Ryvarden, *Pleurotus giganteus* (Berk) Karunarathna & K.D. Hyde, *Hericium erinaceus* (Bull) Persoon, *Schizophyllum commune* (Fr.) and *Ganoderma lucidium* (Curtis) P. Karst. Two methods were used to extract the chemical constituents from these fungi. A hot aqueous extraction protocol was used for the extraction of polysaccharides and proteins, and non-polar constituents were isolated by solvent extraction, using hexane and ethyl acetate as solvents. According to the cytotoxicity assay performed on African green monkey kidney cells, polar extracts were less cytotoxic than non-polar extracts. The aqueous soluble extracts of *Pleurotus giganteus*, *Hericium erinaceus* and *Schizophyllum commune* were less cytotoxic and showed no change in cell viability at concentrations up to 10,000 μg/ml. The strongest anti-dengue activities were found in the aqueous extracts of *Lignosus rhinocerotis* followed by *Schizophyllum commune*, *Hericium erinaceus* and *Pleurotus giganteus*. The highest percentage of inhibitory activity against DENV-2 was observed when the aqueous fungal extract was added at the initial stage of infection.

A recent study investigated the efficacy of plant extracts of *Myristica fatua* Houtt., *Acorus calamus* L. and *Cymbopogon citratus* (DC.) Stapf as antiviral agents against dengue virus infection in human Huh7it-1 cell lines *in vitro* and by molecular docking *in silico*. The *in silico* results were in agreement with the *in vitro* experiments, the extract of *Myristica fatua* contains phytochemical constituents (lignans and acylphenols) that have higher antiviral activities compared to the phytochemical constituents of *Acorus calamus* and *Cymbopogon citratus*. The study revealed that a methanolic extract of *Myristica fatua* reduced DENV infectivity to 21.61% without cytotoxic effect (Rosmalena et al., 2019).

The inhibitory activity of peanut (*Arachis hypogaea* L.) shell extract against influenza virus type A and B has also been studied (Makau et al., 2018). They reported that peanut skin contains high levels of polyphenols (31–34%) which help to prevent lipid overproduction, normalize insulin sensitivity, reduce tissue inflammation, reduce the incidence of liver disorders and infectious diseases, and have antibacterial effects (Rasouli et al., 2017). The inhibitory effects of peanut hull extract are significantly increased when the virus and the extract are added to the cells simultaneously. This suggests that peanut shell extract targets the early stage of viral infection. Peanut hull extract could be used in combination with approved anti-influenza drugs (oseltamivir and zanamivir) to enhance antiviral activity.

*Salvia plebeia* R.Br. (Australian sage) is an edible plant belonging to the *Lamiaceae* family. It has long been used traditionally to treat conditions such as hepatitis, colds, coughs, and hemorrhoids. The chemical composition of *Salvia plebeia* consists of flavonoids, diterpenoids, lignans, caffeic acid derivatives and sesquiterpenoids. It has many biological activities such as antioxidants, hepatoprotectants and antimicrobial activities. Bang et al. (2018) investigated three flavonoids isolated from the methanolic extract of *Salvia plebeia* (6-hydroxyluteolin 7-O-β-d-glucoside, nepitrin and homoplantaginin) using high performance liquid chromatography (HPLC) coupled with diode array detection (DAD) to elucidate the inhibitory activity against H1N1 neuraminidase. The authors concluded that nepitrin exhibited the highest inhibitory activity, followed by 6-hydroxyluteolin 7-O-β-Dglucoside and homoplantaginin. From the chemical structure of nepitrin, it appears that the methoxy group at C-6 and the hydroxyl group at C-5 contribute to the inhibitory activity against influenza neuraminidase.

Honey has been reported to have antiviral activity against the rubella and varicella-zoster viruses and is used to treat recurrent *herpes simplex* lesions. Therefore, scientists tried to find out if honey could be equally effective against the influenza virus. It was found that Manuka honey was effective against the influenza virus among the different types of honey tested - Manuka honey (*Leptospermum scoparium* J.R.Forst. & G.Forst), Soba honey (*Fagopyrum esculentum* Moench; buckwheat), *Potentilla californica* (Cham. & Schltdl.) Greene (honeydew), acacia honey (*Robinia pseudoacacia*) and reindeer honey (*Astragalus sinicus*) - exhibited the highest activity against influenza viruses. The α-ketoaldehyde compound methylglyoxal (MGO) is present in extremely high concentrations in Manuka honey. Recently, MGO has been shown to be the major factor in the antibacterial activity of Manuka honey. It was also observed that combined use of influenza medicines with manuka honey resulted in synergistic effects against influenza virus (Watanabe et al., 2014).

The South African population has been using medicinal plants for decades to treat numerous diseases and life-threatening conditions, such as viral infections. Of the total biodiversity of medicinal plants on South Africa, five of them were investigated as potential antiviral agents against influenza infections due to their beneficial effects on respiratory and inflammatory diseases: *Tabernaemontana ventricose* Hochst. ex A. DC. (wood toad tree), *Cussonia spicata* Thunb. (cabbage tree), *Rapanea melanophloeos* (L.) Mez. (Cape beech), *Pittosporum viridiflorum* Sims (Cape cheese tree) and *Clerodendrum glabrum* E.Mey. (natal glorybower) (Mehrbod et al., 2018). Methanol, ethanol (100 and 30%), acetone, hot and cold water extracts of the powdered plant leaves were tested for antiviral activity against influenza A virus in different combination treatments. The *in vitro* micro inhibition tests revealed that only two of the medicinal plants exhibited significant antiviral activity against influenza A virus, namely *Rapanea melanophloeos* and *Pittosporum viridiflorum*. The methanol extract of *Tabernaemontana ventricosa* showed the least antiviral activity.

### Nanocarriers as Drug Delivery Systems

Although natural products have been shown to have many beneficial pharmacological properties, they have certain limitations in terms of bioavailability and pharmacokinetics. Other limitations include low solubility, poor absorption through the intestinal wall, low hydrophilicity and intrinsic dissolution rate. To effectively address these shortcomings, innovative drug delivery systems tend to be employed, including the use of nanocarriers.

**FIGURE 3:**
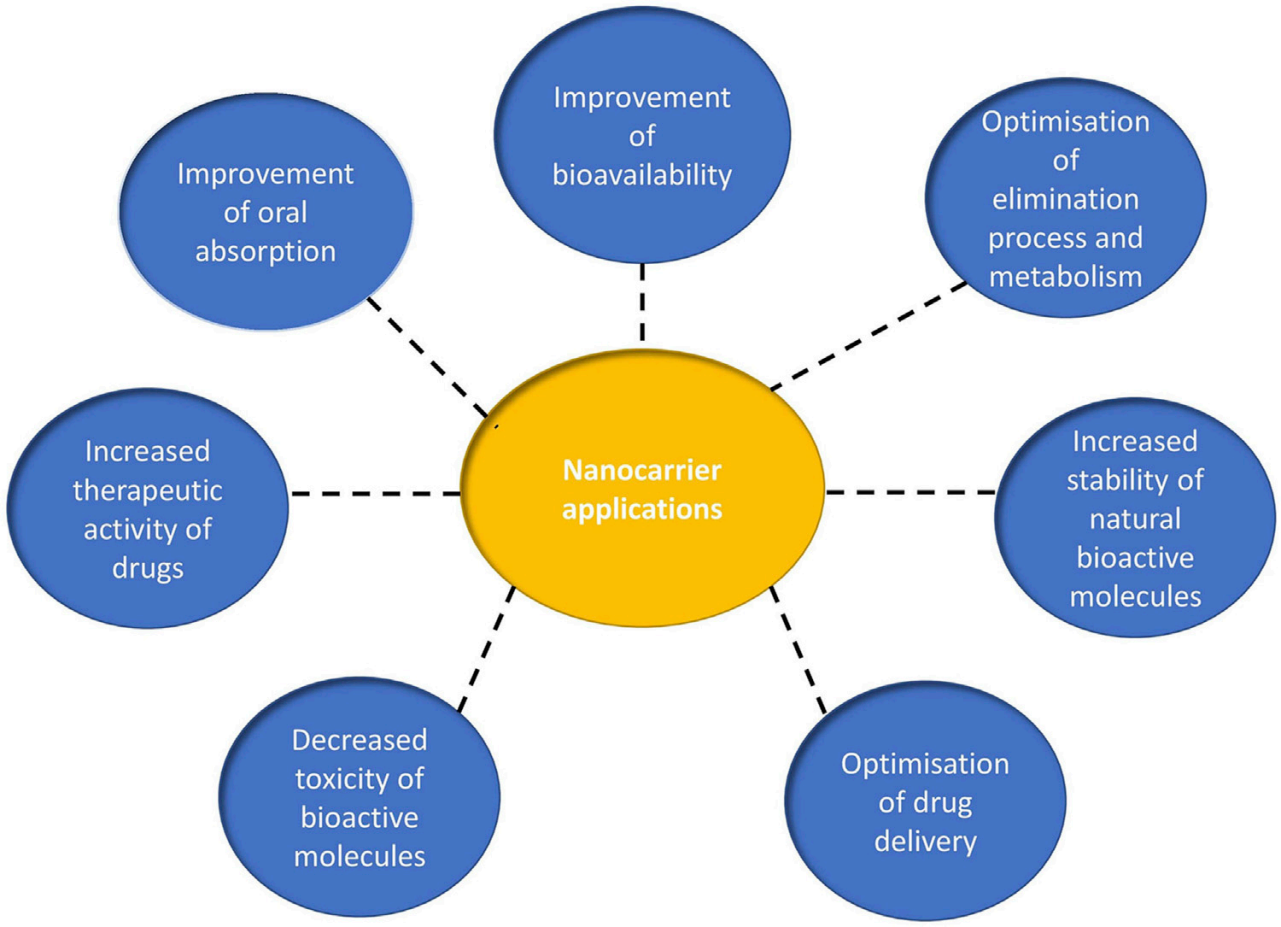
Schematic presentation of the biological activity of nanocarriers as delivery systems for bioactive molecules.

Several nanocarriers have been developed, but few of them are clinically approved for the delivery of specific drugs to their intended sites of action. This chapter is devoted to recent advances in the formulation of various nanocarriers for the encapsulation/conjugation of natural extracts or natural agents with antimicrobial, antiviral and antifungal activity. Nanotechnology and innovative formulations play an important role in improving the stability, bioavailability, efficacy of cellular uptake/internalization, pharmacokinetic profile and reduction of toxicity of the active compounds. A schematic representation of the performances and biological activity of nanocarriers can be seen in Figure 3.

Depending on the structure and formulation method, nanocarriers are classified into the following categories: lipid-based nanocarriers (liposomes, microemulsion drug delivery systems, nanostructured lipid carriers, solid lipid nanoparticles, transfersomes and ethosomes), polymeric nanocarriers (in the form of micelles, nanoparticles, drug conjugates, dendrimers), inorganic nanocarriers and hybrid nanocarriers (Qi et al., 2004; Liu and Feng, 2015; Zhang et al., 2017). This list is constantly being expanded to include novel nanocarriers such as niosomes, liquid crystals and nanotubes (Efferth et al., 2016).

Many such nanocarriers are being tested for efficient delivery of active ingredients extracted from natural sources or even from mixtures and components of various bioproducts. Comparative tests of the efficiency of nanocarriers versus the administration of direct extracts have repeatedly shown the superiority of nanocarriers.

For example, the antibacterial and antifungal activity of various extracts of *Glycyrrhiza glabra* L. was improved by using appropriate nanocarriers adapted to the route of administration. Esmaeili and Rafiee (2014) investigated the antibacterial activity of *Glycyrrhiza glabra* encapsulated in natural polymeric nanoparticles. The preferred ratio of turmeric oil to *Glycyrrhiza glabra* extract was determined to be 10:10 mg/ml based on the effects of particle size and shape. The antibacterial activity of *Glycyrrhiza glabra* extract nanocapsules was evaluated on nine bacterial species. The Gram-positive bacteria tested were *Staphylococcus aureus*, *Enterococcus faecalis*, and *Bacillus cereus*, and the Gram-negative bacteria tested were *Acinetobacter baumannii*, *Pseudomonas aeruginosa*, and *Klebsiella pneumoniae*. The antibacterial activity of *Glycyrrhiza glabra* nanocapsules was superior to *Glycyrrhiza glabra* extract. The antifungal activity was tested on *Glycyrrhiza glabra* L. mucoadhesive nanoparticles containing polylactic acid (PLA), poly(lactic-co-glycolic acid) (PLGA) and alginate embedded in three different mucoadhesive dosage forms: toothpaste, an oral gel and an oral film. Each formulation was tested for its antifungal activity against different *Candida* species (*C. krusei*, *C. guilliermondii*, *C. tropicalis* and *C. glabrata*) (Roque et al., 2014).

In respiratory diseases, the administration of inhalable liposomes loaded with licorice extract (*Glycyrrhiza glabra*) was investigated by Viswanathan et al. (2019) for the treatment of tuberculosis. *In vivo* lung deposition studies of liposomal dry powder for inhalation (LDPI) in mice showed that nearly 46% of the administered drug reached the lungs and 16% of the administered drug remained in the lungs after 24 h of administration. *In vivo* pharmacodynamic evaluation of the LDPI against *Mycobacterium tuberculosis* H37Rv showed a significant reduction in bacterial counts in the lungs and spleen.

Another example of successful delivery of bioactive agents using nanocarriers is artemisinin and its derivatives (e.g., artesunate, artemether, arteether), which exhibit biological activity against protozoa, trematodes, and viruses (Ibrahim et al., 2015). Artemisinin loaded liposomes, nanotubes and fullerenes have been developed and evaluated for their antiproliferative activity for targeted tumor therapy, while artemisinin-based nanostructures lipid carriers and nanoparticles have good anti-malarial properties.

Polymyxin B, produced by the bacterium *Bacillus polymyxa*, belongs to the group of cyclic peptide antibiotics and is used to treat infections caused mainly by Gram-negative bacteria (Chauhan and Bhatt, 2019). Polymixin B cross-linked with sodium alginate and polyion complex nanoparticles or niosomes loaded with polymixin B showed potent antimicrobial activity against *Pseudomonas aeruginosa* (Severino et al., 2015; Insua et al., 2017; Chauhan and Bhatt, 2019).

Another desideratum to be achieved using various nanoformulations is to increase the solubility of the drug. Naringenin (NRG), a flavanone compound, is another drug with a variety of beneficial properties such as antioxidant, anti-inflammatory, antitumor, antifibrogenic, and antiatherogenic. Khan et al. (2015) developed NRG loaded self-nanoemulsifying drug delivery systems (SNEDDS) with the aim of increasing solubility and bioavailability. The increase in drug release and bioavailability compared to drug suspension from SNEDDS formulation can be attributed to the nanoscale droplets and enhanced solubility of NRG in the SNEDDS.

Some nanocarriers respond to pH and allow controlled release in the stomach; for example, since berberine, a benzylisoquinoline alkaloid, has several pharmacological actions, including antimalarial, antiarrhythmic, antihyperglycemic, anticancer, hepatoprotective, antioxidant, and antimicrobial activities (Sahibzada et al., 2018), scientists investigated the biological properties of berberine after encapsulation in different types of nanocarriers, including pH-responsive nanoparticles, dendrimers, and nanoparticles. pH-responsive nanoparticles loaded with berberine showed enhanced suppressive effect on the growth of *Helicobacter pylori* and also reduced gastric inflammation (Lin et al., 2015). Conjugation of berberine with polyamidoamine (PAMAM) dendrimers provided sustained release of the drug after intravenous administration.However, no studies have been conducted on the potential antimicrobial activity of these complexes (Gupta et al., 2017).

Some nanoparticles, such as silver nanoparticles, have intrinsic antibacterial activity that can be enhanced by extracts of plant origin. Garlic (*Allium sativum* L.), for example, has been used as a food and medicinal plant for thousands of years. It has been marketed for its antimicrobial and antiparasitic properties. Robles-Martínez et al. (2019) investigated silver nanoparticles loaded with *Allium sativum* L. against *Trichophyton rubrum*. The results showed a synergistic effect between the silver nanoparticles and the extract. The complex was non-toxic and effective and could provide a safe alternative for the treatment of *Trichophyton rubrum* infections.

The major flavonolignan component isolated from *Silybum marianum* (L.) Gaertn. (milk thistle) is silibinin. This compound has shown liver-protective effects and *in vitro* anti-cancer effects against human prostate adenocarcinoma cells, human breast cancer cells, human ectocervical carcinoma cells, and human colon cancer cells (Garg et al., 2016). Silibinin was encapsulated in phytoliposomes and tested against hepatitis C virus (Ripoli et al., 2016). The loaded phytoliposomes were found to inhibit the binding of the virus to host cells, thus preventing invasion. The phytoliposome/silibilin increased the absorption of silibilin by hepatocytes, thereby increasing its pharmacological activity (three hundredfold) and reducing extrahepatic side effects.

The flavonoid glycosides and crude extracts of *Ginkgo biloba* L. have antimicrobial activity against Gram-positive and Gram-negative bacteria and fungi: *Klebsiela pneumoniae*, *Pseudomonas aeruginosa*, *Staphylococcus* spp., *Streptococcus pyogenes*, *Salmonella enterica*, *Staphylococcus aureus* and *Aspergillus niger*, *Bacillus subtilis*, *Micrococcus roseus*, *Pseudomonas putida, Serratiamarcescens*, *Fusarium oxysporum*, *Thymelaea hirsuta*. (Priyanka et al., 2017; Sati et al., 2018). In a recent study Haghighi et al. (2018) investigated the antimicrobial activity of *Ginkgo biloba* L. solid lipid nanoparticles (SLNs) against two Gram-negative pathogenic strains (*Escherichia coli* and *Pseudomonas aueroginosa*) and one Gram-positive strain (*Staphylococcus aureus*) and found that SLNs from *Ginkgo biloba* extract possessed high antimicrobial activity against all strains studied, and the *in vivo* study showed no short-term toxicity with dermal application of SLNs from *Ginkgo biloba* extract. *Ginkgo biloba* L. extract was also encapsulated in nanoparticles and showed good bioavailability (Wang et al., 2016, 2019).

Green tea extracts contain: (+)-catechin, (−)-epicatechin (EC), (+)-gallocatechin (GC), (−)-epicatechin gallate (ECG), (−)-epigallocatechin (EGC), and (−)-epigallocatechin gallate (EGCG). They also contain a number of natural flavor-intensive components such as terpenes, oxygenated terpenes, sesquiterpenes and organic acids. Gopal et al. (2016) tested particles of green tea extract - nanostores of bioactive catechins - for their bactericidal activity against *Streptococcus mutans* and observed significant antibacterial activity against *Streptococcus mutans* and the human dental bacteria samples from commercial tea. In another recent study, green tea extract was incorporated into liposomes, and this procedure appeared to be favorable for maintaining its antioxidant activity (Dag and Oztop, 2017).

Nano delivery also enhances the antibacterial efficacy of classical antibiotics and antifungals, while reducing their toxicity and allowing repurposing for viral infections. Polymyxin B, produced by the bacterium *Bacillus polymyxa,* belongs to the group of cyclic peptide antibiotics and is used to treat infections caused mainly by Gram-negative bacteria (Chauhan and Bhatt, 2019). Polymixin B cross-linked with sodium alginate and polyion complex nanoparticles or niosomes loaded with polymixin B showed potent antimicrobial activity against *Pseudomonas aeruginosa* (Severino et al., 2015; Insua et al., 2017; Chauhan and Bhatt, 2019).

Amphotericin B is isolated from *Streptomyces nodosus* and is an antifungal drug used for severe fungal infections such as aspergillosis, blastomycosis, candidiasis, coccidioidomycosis, and cryptococcosis and is also a generic drug for leishmaniasis, which is caused by parasites of the genus *Leishmania*. Mehrizi et al. (2018) investigated the effect of amphotericin B encapsulated in nano-chitosan (AK) against *Leishmania major*. *In vitro* results showed that the killing rate of AK was 83% and *in vivo* results showed 80% inhibition of the parasite.

Ivermectin is an antiparasitic chemotherapeutic agent used in both veterinary and human medicine for the treatment of parasitic diseases. It is also used to treat parasitic skin diseases, including scabies and pediculosis. Administration of ivermectin by liposomal formulation against dengue virus resulted in higher *C*max and significantly faster absorption. These findings, obtained through *in vitro* studies, suggest that the use of liposomes could improve the *in vivo* efficacy of ivermectin (Croci et al., 2016).

The most promising antimicrobials encapsulated in different nanocarrier systems and their beneficial effects in terms of biological activity and biocompatibility are summarized in Table 6.

**TABLE 6:** Active substances and nanocarriers used for their enhancement and delivery.

Active substance	Nanocarrier type and size	Formulation method	Biological activity
*Glycyrrhiza glabra* L extract	*Glycyrrhiza glabra* L. **nanocapsules** with natural polymers: chitosan and alginate. Size: 54,3–205 nm	The nanocapsules were obtained through a three-step procedure: o/w emulisification, followed by gelation and solvent removal.	The nanocapsules of *G. glabra* L. showed lower antioxidant activity than the *G. glabra* L. extract in DPPH assay.
			The antibacterial activity of *G. glabra* L. nanocapsules was better than that of *G. glabra* L. extract. Esmaeili and Rafiee (2014)
	*Glycyrrhiza glabra* L. mucoadhesive **nanoparticles** with PLA, PLGA and alginate. Size: 100–900 nm (range)	The extract of *G. glabra* L. was encapsulated in three different polymeric nanoparticles: Alginate, polylactic acid (PLA) and poly(lactic-co-glycolic acid), which were prepared by emulsification/internal gelation and emulsification/solvent diffusion. The encapsulated nanoparticles were embedded in three different mucoadhesive dosage forms: Toothpaste, an oral gel and an oral film.	The antifungal efficacy of each formulation against different *Candida* species (C. krusei, C. guilliermondii, C. tropicalis and C. glabrata) was evaluated.
			These mucoadhesive nanoparticles enhance the absorption of G. glabra L. extract on oral mucosa. Roque et al. (2018)
	**Liposomes** of *Glycyrrhiza glabra* L. (inhalable)	Liposomes containing licorice extract (LE) were prepared by thin layer hydration and freeze dried to produce LDPI (liposomal dry powder for Inhalation).	The LDPI formulation aimed to maximize *in vivo* anti-tubercular activity by administering *Glycyrrhiza glabra* L. directly to the primary site of TB infection – lungs. Viswanathan et al. (2019)
	Size: 210 nm		
Resveratrol	Resveratrol-loaded soy protein **nanoparticles**	Resveratrol encapsulated soy protein isolate (SPI) complexes were prepared by nanoprecipitation method followed by solvent evaporation.	Soy protein nanoparticles loaded with resveratrol showed significantly higher and faster drug release than the free drug. Pujara et al. (2017)
	Size: 100 nm		
Artemisinin	Human serum albumin-bound artemisinin **nanoparticles** (ART/HSA)	The ART/HSA nanoparticles were obtained by precipitation and high-pressure homogenization.	The ART/HSA NPs showed enhanced *in vitro* anti-plasmodial activity. *In vivo* antimalarial activity exceeded that of artesunate in a humanized mouse model. Albumin may provide passive targeting of parasitized erythrocytes Ibrahim et al. (2015)
	Size: mean diameter: 612 nm		
Naringenin (NRG)	**Self-nanoemulsifying** drug delivery system (SNEDDS) of the grapefruit flavonoid naringenin. Droplet size less than 50 nm	NRG was drop wise added in the oily phase by continues stirring.	*In vitro* drug release from SNEDDS was significantly higher (*p* < 0.005) than of the pure drug. Moreover, the area under the drug concentration time-curve (AUC0–24) of NRG from SNEDDS formulation revealed a significant increase (*p* < 0.005) in NRG absorption compared to NRG alone Khan et al. (2015)
		The naringenin and surfactants were mixed for 0.5 h to ensure uniformity.	
		The formulation was equilibrated at room temperature for at least 48 h and examined for signs of turbidity or phase	
		separation prior to dilution, self-emulsification and particle size studies.	
Berberine	pH-responsive Fucose–chitosan/heparin **nanoparticle-**encapsulated berberine	The berberine-loaded fucose-conjugated nanoparticle system was prepared using a freeze-drying process.	The fucose-conjugated NPs with pH-dependent properties can protect berberine from destruction by gastric acids, allowing the drug to penetrate the mucus. The nanoparticles come in contact with *H. pylori* through the carbohydrate fucose receptor. Lin et al. (2015)
	Size: 175.2 ± 5.4; 187.2 ± 6.1; 207.2 ± 7.6 nm		
	**Dendrimer-**berberine nano-conjugates: Amine terminated G4	Berberine was conjugated to amine terminated G4 PAMAM dendrimers by two methods: first one is based on reflux technique and the second one is using microwave irradiation.	Encapsulated and conjugated berberine formulations with PAMAM G4 dendrimer has very good biocompatibility: hemolytic toxicity <5%. Gupta et al. (2017)
	PAMAM dendrimers conjugated to berberine		
	Size: 100–200 nm		
	Berberine **nanoparticles**	Berberine nanoparticles were obtain using evaporative precipitation of nanosuspension (EPN) and anti-solvent precipitation with syringe pump (APSP).	Both NPs present antibacterial and antifungal activity for: *S. aureus*, *B. subtilis, E. coli*, *P. aeruginosa*, *C. albicans, C. glabrata.* Sahibzada et al. (2018)
	Size: 90–110 nm (APSP method) 65–75 nm (EPN method)		
Polymyxin B	Solid Lipid **nanoparticles** (SLN) loaded with Sodium alginate/polymyxin B sulphate SA/PLX) complex	Polymyxin B sulphate (PLX) was cross-linked with sodium alginate (SA) by ionotropic gelation method.	Crosslinking increased the loading of lipid particles with polymyxin and showed greater inhibitory capacity against *Pseudomonas aeruginosa*, which was completely inhibited at all concentrations tested, and SLN-loaded nanoparticles have low cytotoxicity. Severino et al. (2015)
	Size: 439.5 ± 20.42 nm		
	Polymyxin B containing polyion complex (PIC) **nanoparticles**	The Polymyxin B containing PIC nanoparticles was realized using three different polyelectrolyte and a range of degrees of polymerization.	The chemical synthetic strategy was used. The nanoparticles prepared with shorter poly(styrene sulphonate) with low degree of polymerization has the best antimicrobial activity against *Pseudomonas aeruginosa.* Insua et al. (2017)
	Size: 119 nm ± 28%		
	Polymyxin-B (Poly-B)	Polymyxin-B (Poly-B). Niosomes were formulated using Box-Behnken design and thin-film (Cholesterol and Span 60) hydration technique.	Poly-B Niosomes ensure bioavailability as an oral delivery system. The absence of the cytotoxicity and antifungal activity was demonstrated by *in vivo* pharmacokinetic methods. Chauhan and Bhatt (2019)
	**Niosomes**		
	Size: 749.8 ± 73.2–155.3 ± 14.8 nm		
Silibinin	**Phytoliposome-**Based Silibinin Delivery System (PSDS)	Silibinin was extracted from *Silybum marianum* L. Reverse Phase Evaporation (RPE) technique was used to prepare two types of formulations using soybean lecithin (SL) as a common phospholipid matrix. One formulation as control lacking the active guest molecule (simple liposomes, PL) and encapsulated as phytosome (silibininencapsulated phytoliposomes, PS).	Phytoliposome-Based Silibinin Delivery System inhibit hepatitis C virus entry and replication. Also, the natural accumulation of liposomes at the liver level would synergistically increase drug concentration at the target site of action. Ripoli et al. (2016)
	Size: vesicle diameters (PL) 129 ± 3 nm		
	SP) 165 ± 6 nm		
*Ginkgo biloba* extract	*Ginkgo biloba* L. extract (GBE) **nanoparticles- nano-emulsion**	GBE nanoparticles were prepared by the emulsion solvent evaporation method combined with freeze-drying (ESE-FR). The GBE nano-emulsion was prepaired by dissolving the *Ginkgo biloba* extract into the oil phase then the solution was high-speed homogenated. The GBE nano-suspension was obtained afer removing the oil phase.	The percentage of *in vitro* release of GBE nanoparticles was significantly improved compared with that of crude GBE. The plasma concentration of GBE nanoparticles in the rats is much higher than that of crude GBE, and the absorption rate of GBE nanoparticles in the rats is significantly accelerated comparing with that of raw GBE. Wang et al. (2016)
	GBE size: 56.0 nm		
	The particle size of the freeze-dried powder: 277.0 nm		
	*Ginkgo biloba* L. extract (GBE) loaded **solid lipid nanoparticles** (SLNs) Size: 104–621 nm	GBE-loaded solid lipid nanoparticles were synthesized by using high pressure homogenization method.	GBE/SLNs present antimicrobial activity against *E. coli*, *P. aueroginosa* and *S. aureus.*
			*In vivo* cytotoxicity tests using female Albino rabbit (OECD 404 Guideline) didn’t show any toxicity, respectively no irritant or corrosive effect for short and long term from dermal application.
			(Pegah et al., 2018)
	*Ginkgo biloba* L. extract **nanoparticles** Size: 76.90 nm	*Ginkgobiloba* L. extract nanoparticles were prepared by liquid anti-solvent precipitation (LASP)	The bioavailability of the nanoparticles was better than that of raw GBE, in which the AUC(_0->t_) value of flavonoids in GBE nanoparticles
			were 2.20 times of that in raw GBE. Wang et al. (2019)
Green tea extract	Natural **particles** of green tea extract - nano stores of the bioactive catechins	Green tea particles suspended in the green tea extract were separated by differential centrifugation, forming three categories according to size range: Macrosized tea particles, Microsized tea particles and finally Nanosized tea particles	The antimicrobial activity is due to presence of catechins polyphenols which damage the bacterial cell membrane. Gopal et al. (2016)
	Size: 50 nm–80 μm		
	**Liposomes** Green tea extract	Encapsulation of the green tea extract was achieved by dispersing 1% (w/v) soy lecithin by high pressure homogenization (microfluidization).	Encapsulation of green tea extract in liposomes is a promising technique to protect the antioxidant activity of the extract, with possible applications in food industry as a functional food ingredient. Dag and Oztop (2017)
	Size: 50–120 nm (microfluidization)		
	Size: 70–130 nm (ultrasonication)		
Amphotericin B	**Nano-sized** chitosan amphotericin B	Nano-sized chitosan amphotericin B synthesized by drug adsorption and phase separation methods	Bioactivity against *Leishmania* parasites was tested and higher cellular uptake was observed. In terms of efficacy against the parasite, an inhibition of 83% was observed, with both promastigote and amastigote being achieved without any toxicity due to the use of a higher effective dose. Mehrizi et al. (2018)
	Size: 102–112 nm		
Ivermectine	Liposomal **nanocarriers** Ivermectine	Liposomes were prepared by a modification of the ethanol injection method, in which the ethanolic solution of lipids is injected into the water phase at a controlled rate.	Ivermectin liposomes have been tested against dengue virus replication. Cytotoxicity is up to 5 times lower when ivermectin is administered through liposomes. The increase in antiviral activity (measured as EC_50_ on DENV 2) from 2.6 to 0.3 µM is due to the fact that liposomes containing ivermectin can fuse with cell membranes, facilitating intracellular uptake of the drug. Croci et al. (2016)
	Size		
	Neutral liposomes: 65–193 nm		
	Cationic liposomes: 30–72 nm		
*Allium sativum* L extract	*Allium sativum* L. extract/silver nanoparticles (AgNPs) **complex.** Size of Silver NPs: 26 ± 7 nm	AgNPs synthesis was performed using a modified Lee-Meisel seed synthesis method. Decoration of the extract of *Allium sativum* L. with AgNPs was carried out by homogenising the AgNPs with the extract in an aqueous solution.	The complex of *Allium sativum* L. extractand silver nanoparticles (AgNPs) was tested for its antifungal activity against *Trichophyton rubrum*.
			The combination of *Allium sativum* L. extract with AgNPs proved synergistic and enhanced the activity (Martínez et al., 2019)

## Discussion

One of the recently discovered bacteriocins, *Plantacyclin* B21AG, appears to have excellent stability and bactericidal activity against sporulating bacteria such as *Clostridium perfringens* and non-sporulating *Listeria monocytogenes*. This makes it an important candidate not only for food preservation but also for effective therapeutics against foodborne diseases. Among the plant compounds with antimicrobial activity against Gram-positive bacteria, curcumin, essential oils of coriander, cumin, mustard seed and oregano were the most effective, while resveratrol also exhibited antibiofilm properties. Plant extracts were intensively tested for their antibacterial activity against Gram-negative strains, including ethanolic extract of *Chimaphila umbellata* (L.) W.P.C.Barton, *Hypericum roeperianum* G.W. Schimp. ex A.Rich., acetone extracts of *Maesa lanceolata* Forssk, and methanolic extract of *Oxalis corniculate* L. Recently studied compounds like pectins and pelargonic acid also showed good bactericidal activity on *Salmonella* spp. strains. The essential oil extracted from *Cinnamomum zeylanicum* flower was very effective against *Pseudomonas* spp. with very low MIC values.

Newly described antimicrobial agents of animal origin are arenicins and seroins. Arenicins had impressive antimicrobial activity against Gram-negative XDR and MDR strains, while seroins appear to have stronger bacteriostatic activity against Gram-positive strains.

By far the most impressive bacteriocin is the newly discovered enterocin 12A, isolated from *Enterococcus faecium*, which not only inhibits MDR Gram-negative strains but also blocks the proliferation of cancer cells.

Most natural antimicrobial agents that target bacteria appear to disrupt membrane permeability, leading to membrane rupture and cell lysis. However, not all mechanisms of action have been elucidated, and sometimes the mechanism may be indirect, stimulating the host immune system or inhibiting adhesion to the host cell.

Among the various plant extracts studied, *Eucalyptus globulus* Labill, *Cinnamomum verum* J. Presl and *Buchenavia tomentosa* Eichler have been found to have good antifungal activity against *Candida albicans*. *Scutellaria baicalensis* Georgi root extracts showed marked antifungal activity against some dermatophytes, *Aspergillus fumigatus* and *Candida albicans*. Intraperitoneal infusions of gallic acid increased the cure rate in mice with systemic *Candida albicans* infection. Microorganism-derived secondary metabolites such as meijiemycin and jagaricin have shown promising antifungal activity against fungal strains. Bioactive molecules from natural sources exert their antifungal effects through a variety of mechanisms, such as those currently targeted by antifungal drugs used in the clinic, but also through novel mechanisms, such as altered DNA/RNA function, accumulation of ROS or cell cycle disruption. The study of natural product libraries or the activation of silent genes in microorganisms and plants is a promising pipeline for new compounds with antifungal activity. In addition to actual antifungal activity, many of the molecules derived from natural sources, such as monoterpene phenols, have been shown to enhance the effects of currently used antifungal drugs.

Recent studies show that some plant extracts (phytochemicals) such as phenols from peanut shell extract and flavonoids from mulberry species and *Salvia plebeia* R.Br. extract have a significant inhibitory effect on virus replication. Complex mixtures such as Manuka honey and extracts of *Veronica persica* Poir. exhibit antiviral properties and could be used in combination with antiviral drugs to improve treatment selectivity and efficacy.

Nanocarriers improve pharmacokinetics, stability and solubility, reduce toxicity and provide controlled release of therapeutic agents or other compounds at the target site. Recently, nanoparticles extracted from natural products such as *Glycyrrhiza glabra* L. extract, green tea extract, *Allium sativum* L. extract and *Ginkgo biloba* L. extract have been shown to have potent antimicrobial properties, while some lipid carriers such as silibinin phytoliposomes, artemisinin liposomes and ivermectin liposomes have good antiviral activity. Nanocapsules from *Glycyrrhiza glabla* L., polymixin B cross-linked with sodium alginate and solid lipid nanoparticles from *Ginkgo biloba* L. showed potent antimicrobial activity against *Pseudomonas aeruginosa*. In addition, a possible treatment for fungal infections could be the use of *Allium sativum* extract encapsulated in silver nanoparticles, Polymyxin B niosomes and *Glycyrrhiza glabra* L. mucoadhesive nanoparticles with PLA, PLGA and alginate.

Drug delivery systems have evolved greatly in recent years, and a wide range of nanocarriers are available to researchers to enhance the biological activity of natural products. However, there is a great need for in-depth research if a nanocarrier-based drug delivery system and extracts of natural products are to be matched. Research teams must achieve the desired biological effect and ensure that there is high compatibility between the nanocarrier and the biologically active molecule, while minimizing adverse effects such as cytotoxicity.

Many of the currently available studies rely on *in silico* and/or *in vitro* testing as a preliminary step in determining the health benefits of natural products with or without the use of a carrier system. The *in vitro* tests may reveal a potential antimicrobial effect of the natural product/natural product-delivery system complex, but these results need to be complemented by toxicity tests to avoid serious side effects, followed by *in vivo* studies, using the most suitable animal models to determine whether specific compounds in the body may inhibit, block, degrade or interfere with the drug.
